# Phylogenetic systematics of *Schacontia* Dyar with descriptions of eight new species (Lepidoptera, Crambidae)

**DOI:** 10.3897/zookeys.291.3744

**Published:** 2013-04-17

**Authors:** Paul Z. Goldstein, Mark A. Metz, M. Alma Solis

**Affiliations:** 1 Department of Entomology, University of Maryland, College Park, MD USA; 2 National Museum of Natural History, E-523, P.O. Box 37012, MRC 168, Washington, DC 20013-7012; 3 Systematic Entomology Laboratory, USDA, National Museum of Natural History, P.O. Box 37012, MRC 168, Washington, DC 20013-7012

**Keywords:** *Schacontia*, Crambidae, Glaphyriinae, Brassicales, secondary sexual characters

## Abstract

The Neotropical genus *Schacontia* Dyar (1914) is reviewed and revised to include eleven species. *Schacontia replica* Dyar, 1914, **syn. n.** and *Schacontia pfeifferi* Amsel, 1956, **syn. n.** are synonymized with *Schacontia chanesalis* (Druce, 1899) and eight new species are described: *Schacontia umbra*,**sp. n.,** *Schacontia speciosa*,**sp. n.,** *Schacontia themis*, **sp. n.,** *Schacontia rasa*, **sp. n.,** *Schacontia nyx*,**sp. n.,** *Schacontia clotho*, **sp. n.,** *Schacontia lachesis*, **sp. n.,** and *Schacontia atropos*, **sp. n.** Three species, *Schacontia medalba*, *Schacontia chanesalis*, and *Schacontia ysticalis*, are re-described. An analysis of 64 characters (56 binary, 8 multistate; 5 head, 13 thoracic, 13 abdominal, 25 male genitalic, and 8 female genitalic) scored for all *Schacontia* and three outgroup species (*Eustixia pupula* Hübner, 1823, *Glaphyria sesquistrialis* Hübner, 1823, and *Hellula undalis* (Fabricius, 1781)) retrieved 8 equally most parsimonious trees (L=102, CI=71, RI=84) of which the strict consensus is: [[[[*medalba* + *umbra*] + *chanesalis*] + speciosa] + [*ysticalis* + [*rasa* + *themis* + [*atropos* + *lachesis* + *nyx* + *clotho*]]]]. The relevance of male secondary sexual characters to the diagnosis of *Schacontia* species is discussed.

## Introduction

**Figures 1–12.**
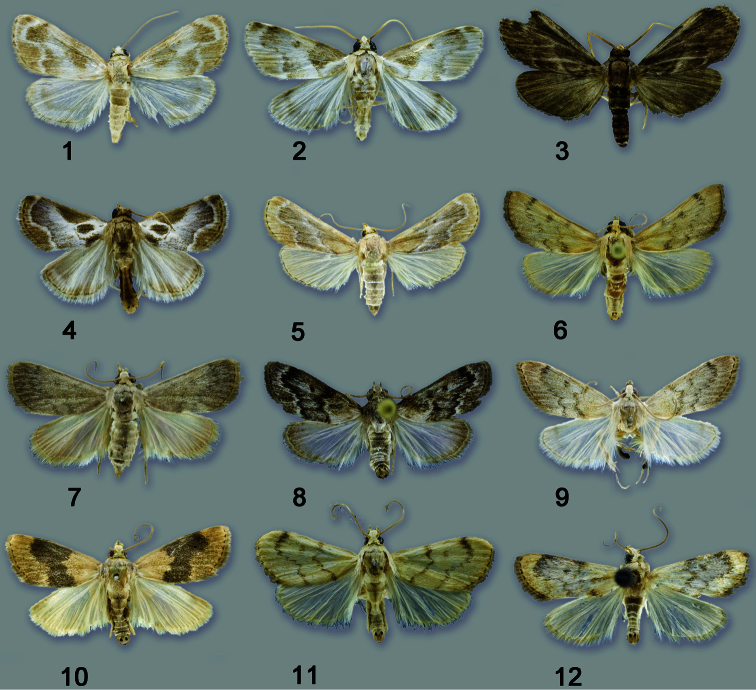
Habitus of adults. **1** *Schacontia medalba* male, Peru, 1883, “Boniti P., Peru, Jan. 7. 83” **2** *Schacontia chanesalis* male, Mexico, Becker 68741, Tam El Ensino 250 m, 4–13.viii.1988, V.O.Becker Col. **3** *Schacontia umbra* male paratype, “Col. Becker 100503, Ecuador: Past. Mera, 1300 m, xii 1992, V.O. Becker Col, *Schacontia* n. sp. #3 det. M.A. Solis” **4** *Schacontia speciosa* male holotype, “Col. Becker 65271, Brasil, RJ Marica, 5 m, 11.x.1985, V.O. Becker Col. **5** *Schacontia ysticalis* Sirena, Corcovado Nat. Pk., Osa Penin., Costa Rica, 19–27 Mar 1981, DH Janzen, W. Hallwachs **6** *Schacontia themis* Venezuela, Guarico, Hato Masaguaral, 45 km S Calabozo, 8.57N, 67.58W, Galry For #20, 75 m, 13–16 May 1988, uv lt., M. Epstein & R. Blahnik **7** *Schacontia rasa* male holotype, Col. Becker 110514, Mexico, Tam San Fernando, 50 m, 28.vi.1997, V. O. Becker Col. **8** *Schacontia nyx* “Venezuela: Guarico, Hato Masaquaral, 45 km S Calabozo, 8.57N, 67.58W, Galry Forest #20, 75 m, 13–16 May 1988, uv lt., M. Epstein & R. Blahnik **9** *Schacontia clotho* male holotype, Col. Becker 102660, Ecuador, Loja Catamayo 1300 m, 20.xii.1992, V.O. Becker Col., Genitalia 1287 **10** *Schacontia lachesis* male, “Col. Becker 55439, Brasil, RJ Arrai al do Cabo, 50 m, 29.i.1985, V.O. Becker Col.” **11** *Schacontia lachesis* male, “Bolivia, Santa Cruz, Puerto Suarez, 150 m, Nov 1908, J. Steinbach, CMNH Acc. 3758” **12** *Schacontia atropos* male holotype, “San Estaban, Carabobo, Venez., Dec. 1–20 1939, Pablo J. Anduze.”

*Schacontia* Dyar, 1914: 400 represents a small cluster of species recently transferred to the Glaphyriinae (Solis 2009) (Figs 1–12). Both the male genitalia and the external appearance of described *Schacontia* are atypical for pyraloids, so much so that the type species was originally described by Schaus as a member of the noctuid genus *Acontia* Ochsenheimer (as “*Acontia*? [sic] *medalba*”; Schaus, 1904: 163). The subsequent taxonomic history of these moths is one of taxonomic curiosity and nomenclatural flux. *Schacontia* caught the attention of taxonomists in part by virtue of its unusual male genitalic apparatus, which comprises a uniquely configured gnathal complex and reduced valvae. *Schacontia* wasoriginally described in the Schoenobiinae, retained there by Amsel (1956), transferred to the Epipaschiinae (Pyralidae) by Munroe (1958), and then tentatively transferred to the Cybalomiinae (Munroe 1995). It was most recently transferred to the Glaphyriinae by Solis (2009) based in part on the external morphology and genitalia, but mostly based on the morphology of the tympanal organs. Solis (2009: 493) characterized the subfamily with the following combination of characters: chaetosemata absent; concavity on the costa of the forewing present; fovea between Rs_2+3_ and Rs_4_ present; forewing with Rs_4_ in a non-apical position and a costal crescent present; and lateral indentations of Sternite 2 present (Luquet and Minet 1982).

With respect to their actual biology, *Schacontia* larvae have been variously associated with Capparaceae (Brassicales) and have been recently reported as parasites of cassidine chrysomelid beetles (Cuignet et al. 2008), but that latter report is unverified as *Schacontia*. Without more life history data and more taxon-rich analysis, it is not possible at this time to address life history evolution, the macroevolution of host plant associations, or larval feeding behaviors in *Schacontia*. It is in the widespread species (*Schacontia chanesalis*, *Schacontia themis*, *Schacontia ysticalis*) that life history and larval data are most sorely needed rangewide. The association of some species with Capparaceae is not unusual for crambids (cf. Solis et al. 2009), and as such, *Schacontia* may provide a forum for exploring its origin(s). The evolution of glucosinolates in the Brassicales (e.g., Mithen and Marquez 2010) may bear on the origins of these moths’ specialized feeding habits, including the gall-forming behavior and internal feeding reported by Solis et al. (in prep.).

In the present work, we treat newly assembled historical and recent material from the Western Hemisphere. Our purpose is to refine the circumscription and composition of *Schacontia* by identifying and describing new species and presenting a phylogenetic analysis of their relationships. Recent collecting and rearing work, including the efforts in Costa Rica by D. Janzen and W. Hallwachs, have generated life history information, most importantly the association of *Schacontia* with capparaceous plants. Those potentially allied with *Schacontia* on the basis of wing venation and features of the gnathos and tegumen, comprise eight undescribed species ranging from Mexico through Central and South America and the Caribbean, some narrowly endemic, others widespread.

## Materials and methods

Pinned specimens were examined with an incandescent light source (reflected light). Male and female genitalic preparations varied, some of those pre-dating this study having accumulated from several sources. Most were prepared following Clarke (1941), using chlorazol black and in some cases mercurochrome as staining agents; Eosin-Y was used in some preparations [those originating with Dr. V.O. Becker]. The more recent dissections were made following a hot soak in supersaturated sodium hydroxide, and held in glycerine caps or temporary slides for character scrutiny. Some older preparations of wings were prepared following Borror et al. (1989): soaked in bleach, stained with Eosin-Y, and slide mounted in Canada balsam. Slide preparations were examined with dissecting and compound microscopes. Photographs were made using the Microptics and Visionary Digital imaging systems and images manipulated with the Gnu Image Manipulation Program (The GIMP Team, gimp.org) and, when appropriate, retouched with Adobe Photoshop® (Adobe Systems, Mountain View, CA). All measurements were made with the aid of an ocular micrometer. Forewing length was measured from the center of the axillary area up to the apex of the forewing (FW). Terminology follows Wooton (1979), Klots (1970), Maes (1985, 1995), Yoshiyasu (1985), Phillips and Solis (1996), Solis and Maes (2002), and Mally and Nuss (2011), except where noted (Figs 63, 64). The terminology of Maes (1985, 1995) is adhered to strongly with respect to the tympanal organs; Mally and Nuss (2011) are consulted as a more recent reference with respect to coding the female genitalic characters.

### Material examined

This work drew in part from an effort to treat taxa with taxonomic and nomenclatural problems identified during preliminary surveys of pyraloids in the extensive Costa Rican collection of D. Janzen and W. Hallwachs. Because the genitalic characters of *Schacontia* species had not been adequately explored, specimens of all known Costa Rican species were initially dissected to survey diagnostic characters of each species and putative synapomorphies for the genus. In order to determine the nomenclatural status of Costa Rican species, types of all neotropical species at the Zoologische Staatssammlung München, Munich, Germany (ZSM), Naturhistoriches Museum, Vienna (NHMV), The Natural History Museum, London (BMNH), and the National Museum of Natural History, Smithsonian Institution, Washington, DC (USNM) were examined. Following this preliminary work, a more expansive series of material (all the specimens of *Schacontia* we could locate) was examined, most recently including Bolivian and Puerto Rican material housed at the Carnegie Museum of Natural History (CMNH) as well as at Cornell University Insect Collection (CUIC), and all the available material at USNM, including material from the V.O. Becker collection (VOB). Specimens are listed for each species with all attendant label data, including genitalic dissection slide numbers and record numbers from the database of Janzen and Hallwachs (http//Janzen.sas.upenn.edu). Primary types are deposited at the USNM (Washington, DC) and the CMNH (Pittsburgh, PA).

### Repository abbreviations

The following abbreviations refer to collections from which specimen material forms the basis of this work:

**BMNH **The Natural History Museum [statutorialy: British Museum (Natural History)], London, UK

**CMNH **Carnegie Museum of Natural History, Pittsburgh, PA, USA

**CNC **Canadian National Collection of Insects, Arachnids, and Nematodes, Ottawa, Ontario, Canada

**CUIC **Cornell University Insect Collection, Ithaca, NY, USA

**INBio** Instituto Nacional de Biodiversidad (INBio), Santo Domingo de Heredia, Costa Rica

**MGCL** McGuire Center for Lepidoptera and Biodiversity, Florida Museum of Natural History, University of Florida, Gainesville, FL, USA

**NHMV** Naturhistoriches Museum, Vienna, Austria

**USNM **National Museum of Natural History [formerly, United States National Museum], Washington, District of Columbia, USA

**ZMHB **Museum für Naturkunde der Humboldt-Universität, Berlin, Germany

**ZSM** Zoologische Staatssammlung, Munich, Germany

Following the identification of a suite of male secondary sexual characters suspected of diagnosing multiple species pairs, we undertook a preliminary DNA barcoding exploration of two putative species for which relatively recent (~20 year old) material existed. Sequencing was done using standard protocols at the Biodiversity Institute of Ontario (Hrcek et al. 2011; Wilson 2012).

### Character coding and cladistic analysis

All characters were equally weighted and coded as unordered. Preference was given to combinations of binary and inapplicable coding schemes over multistate characters. Conspecificity of male and female specimens was inferred by locality when other biological information was unavailable. Preliminary phylogenetic analysis involved parsimony searches via the ratchet routine (island-hopper, 1000 iterations per rep) in Windada/Winclada (1000 iterations per rep; Nixon 1999-2002). Character selection and coding shemes were reevaluated repeatedly following successive rounds of tree search as the matrix was developed. The Winclada (.winc) file was re-saved as a NONA (.ss) file and then as a TNT file, using a text editor to ensure rooting at the first terminal encountered. Exhaustive searches (implicit enumeration) were then run in TNT (Willi Hennig Society edition; Goloboff et al. 2008). Bremer values (Bremer 1988) were calculated in TNT from exhaustive searches of progressively longer suboptimal trees (increments of 1 step). Synapomorphies were mapped with Winclada.

## Systematics

### Taxonomic scope and outgroup selection

The scope of our treatment of described *Schacontia* is based on Munroe (1995); type material is deposited at USNM except where designated in text. We treat the taxonomic and nomenclatural issues in the subfamilial placement of the genus only insofar as they pertain to outgroup selection and rooting.

Initial examination of specimens tentatively identified as *Schacontia* revealed, first, a cohesive group of species comparable to the type species [*Schacontia medalba* (Schaus, 1904)] unified by a uniquely hood-like or mucronate uncus, reduced male valvae, a divided tegumen with a prominent medial sulcus, and a gnathos with a unique, four-armed configuration. A somewhat more variable group, including *Schacontia ysticalis* (Dyar, 1925) and several undescribed species, appeared to bear similarities to *Schacontia* in forewing pattern and, in modified form, features of the male valva, tegumen, and gnathos. Bearing in mind that member species of *Schacontia* have been placed in several subfamilies prior to the genus’ transfer to Glaphyriinae by Solis (2009), and in the interest of being thorough, type species of all 33 known glaphyriine genera were examined as outgroup candidates. We also surveyed types of *Cybalomia* Lederer, 1863 and a range of crambid subfamilies in order to mine specific character systems for putative synapomorphies of *Schacontia* and to ensure proper character polarization. We selected three glaphyriine outgroup taxa, all type species of their respective genera: *Eustixia pupula* Hübner, 1823, *Hellula undalis* Fabricius, 1781, and *Glaphyria sesquistrialis* Hübner, 1823, at which our tree is rooted. These outgroups were chosen both on the basis of their status as name-bearers and on the basis of what we estimated to be comparable suites of observable similarities (putative homologies) with *Schacontia medalba*. The rooting at *Glaphyria sesquistrialis* was implied by the current classification, but more importantly was based on a preliminary screening of male and female genitalia and tympanal structures. These include the configuration of the saccus tympani and corpus bursae.

A total of 64 characters (56 binary, 8 multistate; 5 head, 13 thoracic, 13 abdominal and tympanal, and 25 male genitalic, and 8 female genitalic) were adduced and coded (below; see Appendix I for the full matrix). Inapplicable and missing data were coded with “-” and “?”, respectively.

**Figures 13, 14.**
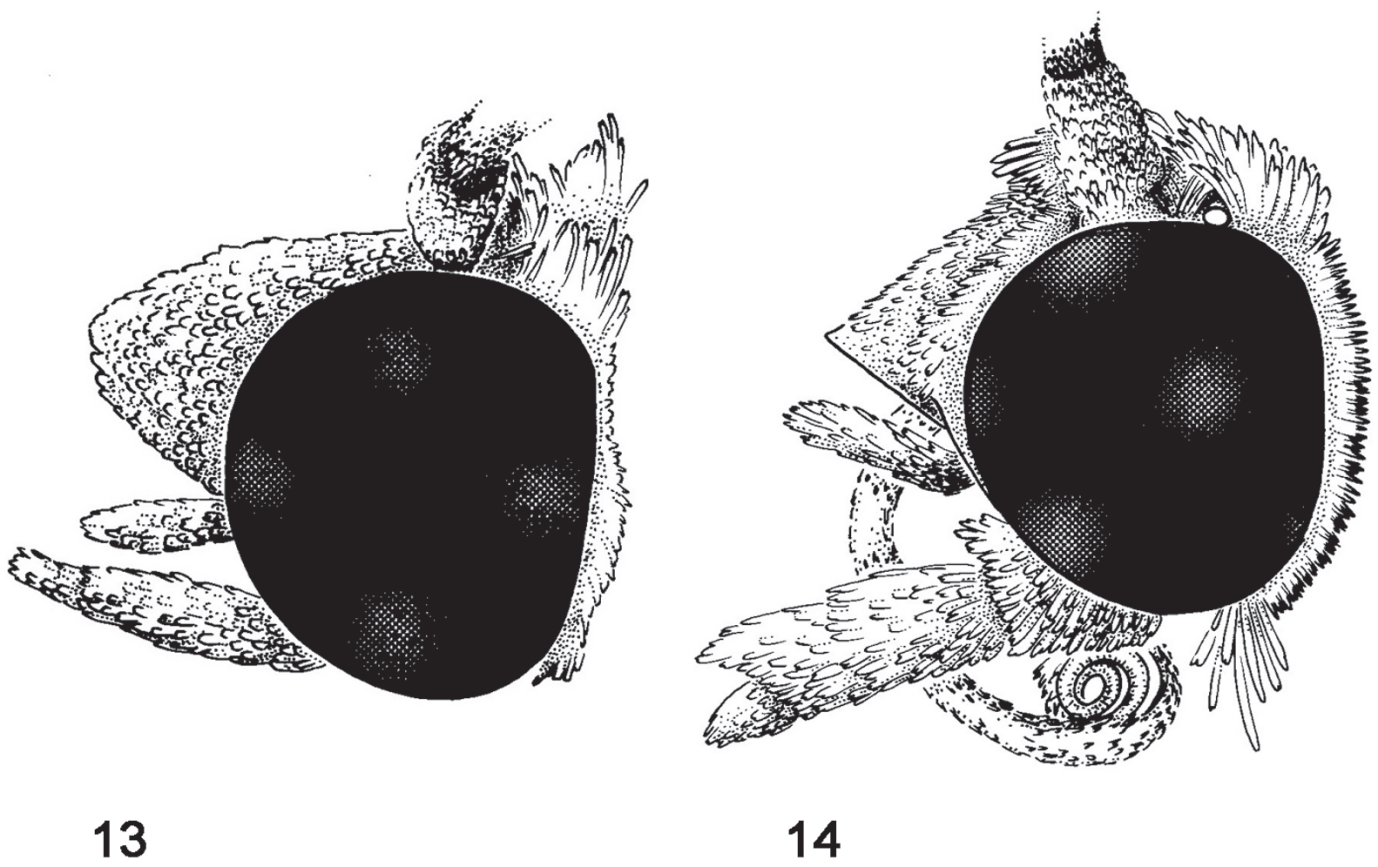
Head, lateral view. **13** *Schacontia chanesalis*; frons “normal” **14** *Schacontia ysticalis*; frons carinate.

**Head [Figs 13, 14]**

0. Ocelli: (0) absent; (1) present

1. Proboscis: (0) reduced, inconspicuous; (1) conspicuous (Figs 13, 14)

2. Frons: (0) of normal contour, evenly rounded; (1) conical or expressed as a small hump; (2) carinate or otherwise modified (Figs 13, 14)

3. Length of labial palpus: (0) extending beyond clypeus; (1) not extending beyond clypeus

4. Maxillary palpi: (0) extending anteriorly beyond frons; (1) not reaching anterior margin of frons

**Figures 15–17.**
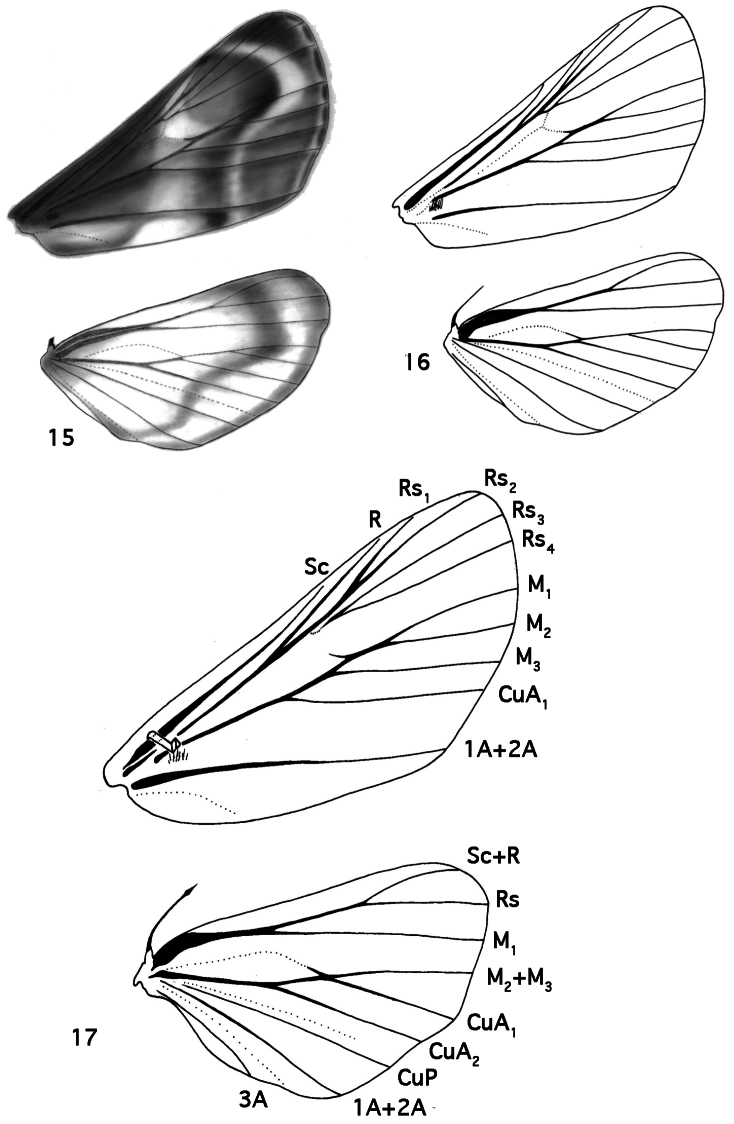
Wings. **15** *Schacontia chanesalis* female underside **16** *Schacontia chanesalis* female underside **17** *Schacontia chanesalis* male underside.

**Figures 18–20.**
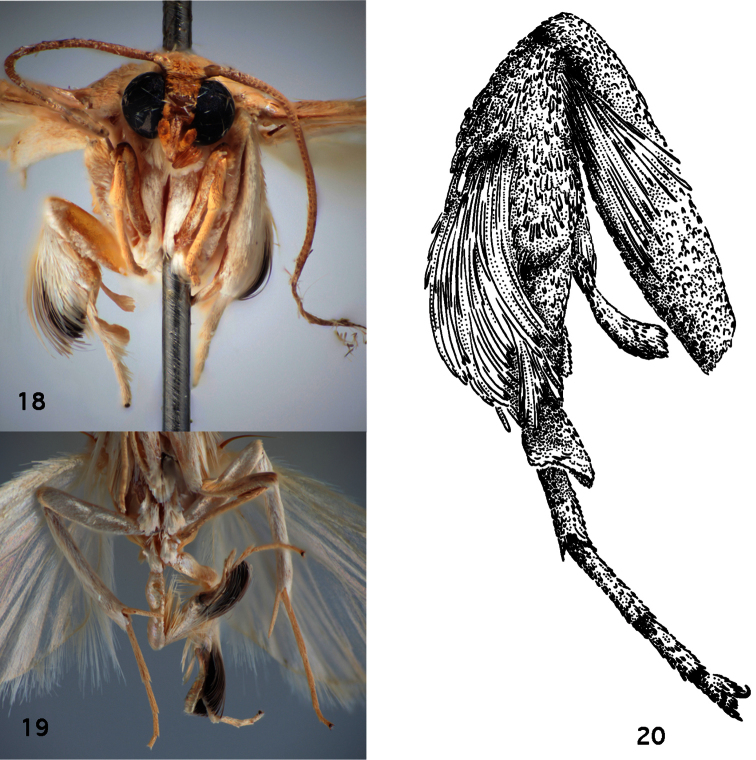
Thoracic and leg structures in *Schacontia clotho* and *Schacontia themis*. **18** *Schacontia themis*, hind leg, frontal view, illustrating secondary sexual characters: flattened hind tibial spur, scales with dark patch, and flattened concave metatarsal modification, and epipleural setae (data Fig. 6) **19** *Schacontia clotho*, ventral view, illustrating darkened hind tibial scales. Ecuador: Loja Catamayo, 1300 m, 20.xii.1992, V.O. Becker Col; Col. Becker 102660 **20** *Schacontia themis*, hind leg, lateral view.

**Thorax [Figs 15**–**20]**

5. Forewing (Rs_3_, Rs_4_): (0) bases separate; (1) stalked (Figs 16, 17)

6. Forewing (M_1_, M_2_): (0) bases separate; (1) stalked (Figs 16, 17)

7. Medial area: (0) contrast with basal and postmedial areas subtle, almost unicolorous except for lines, spot; (1) contrast between medial area and basal and postmedial areas sharp

8. Forewing coloration: (0) compound, ground color not uniform in any given area (antemedial, medial, postmedial; Fig. 15); (1) two-toned, medial area contrasts with basal and apical area in ground color

9. Concentration of white scales apical to antemedial line: (0) absent, or if present then only diffusely; (1) present (Fig. 15)

10. Hindwing (HW) postmedial line: (0) not conspicuous or nearing inner margin; (1) distinct, approaching or reaching inner margin (Fig. 15)

11. Distance between postmedial line and wing terminus: (0) narrow (Fig. 15); (1) wide

12. Wing lines: (0) dark on light ground; (1) light on dark ground (Fig. 15)

13. HW (M_2_M_3_+CuA_1_): (0) bases separate; (1) stalked (Figs 16, 17)

14. Male hind leg secondary sexual complex consisting of a flattened hind tibial spur with flattened scales and basal tarsus with concave spoon-like modification: (0) absent; (1) present (Figs 18–20)

15. Dark patch amidst hind tibial scales: (0) absent; (1) present (Figs 18, 19)

16. Tufts of epipleural setae: (0) absent; (1) present

17. Female medial hind tibial spurs: (0) one pair (medial pair absent); (1) two pair (medial pair present)

**Abdomen - Tympanal characters [Figs 21–32]**

18. Bullae tympani invaginated in Sternum 2: (0) not (all ingroup taxa; Figs 21–32); (1) slightly to strongly

19. Saccus tympani invagination: (0) short, not beyond puteolus; (1) deep, with posterior ridge, but not prominently invaginated posteriad (Figs 21–23); (2) capacious, ovate chamber; conspicuous broad lip (Figs 24–32)

20. Saccus: (0) not prominent (Figs 21–23); (1) prominent (Figs 24–32)

21. Mesal extent of saccus (@ pons): (0) short (Figs 21–23); (1) intermediate (Figs 24–32);

22. Puteoli: (0) absent or indistinguishable from saccus tympani (all ingroup taxa; Figs 21–32); (1) present, if small

23. Processus tympani: (0) inconspicuous; (1) approximately semi-circular (Figs 21–23); (2) thumblike, lobulate (Figs 24–32)

24. Fornix, protrusion over venula prima: (0) protruded over slightly, flat; (1) far removed from edge (all ingroup taxa; Figs 21–32)

25. Fornical ulna: (0) > 90 degrees or low arc (all ingroup taxa; Figs 21–32); (1) < 90 degrees

26. Sclerotization of fornix: (0) light to moderate; (1) heavy (all ingroup taxa; Figs 21–32)

27. Fornix: (0) robust, broad (Figs 21–24); (1) narrow, ribbonlike (Figs 24–32)

28. Venulae secundae: (0) wide, gently tapered (Figs 21–24); (1) more sharply tapered, elongate, forming a neck (Figs 24–32)

29. Tergo-sternal sclerite: (0) present, not elongate (Figs 21–24); (1) prominent, elongate, roughly equivalent in length to that of bulla tympani (Figs 24–32)

**Abdomen - Post-tympanal characters [Figs 33–35]**

30. Coremata on 4^th^ abdominal segment: (0) absent; (1) present (Figs 33–35)

**Male genitalia [Figs 36–65, part]**

31. Gnathos-ventrotergal rods complex: (0) absent; (1) present (all ingroup taxa; Figs 36–65, in part)

32. Gnathos, middle process: (0) absent; (1) present (all ingroup taxa; Figs 36–65, in part)

33. Dorsal ridges of tegumen: (0) absent, split to uncus, or inverted U; (1) cruciate, crossing near uncus (Figs 46, 49, 52, 55, 58, 61, 64); (2) inverted upsilon with medial ridge (Figs 36, 39, 42, 44)

34. Teguminal sulcus: (0) absent; (1) present (all ingroup taxa; Figs 36–65, in part)

35. Uncus tip trefoil shaped: (0) absent (Figs 36, 39, 42, 44, 46); (1) present (Figs 49, 52, 55, 58, 61, 64)

36. Shape of trefoil, if present: (0) reduced, rhomboid (Figs 55, 58, 61, 64); (1) expanded, hastate (Figs 49, 52)

37. Uncus edges: (0) simple, undifferentiated (Figs 36, 39, 42, 44, 46, 55, 58, 64); (1) modified, swollen (Figs 49, 52, 61)

38. Uncus, interior (under-surface: (0) clear, without relief (Figs 36, 39, 42, 44, 46, 55, 58, 64); (1) with elongate central development appearing as a raised ridge (Figs 49, 52)

39. Valva - outer margin: (0) entire or emarginate, but continuous, such that trajectory of valval membrane continues apically (Figs 36, 39, 42, 44); (1) trajectory of valval membrane recurves such that upper and lower extensions are evident, a fleshy lobe bearing a setal tuft associated with end of costa, and the subcostal projection sclerotized dorsally (Figs 46, 49, 52, 55, 58, 61, 64)

40. Glabrous central area of valva: (0) Absent [valva elongate, setose]; (1) truncate - squared or subquadrate (emarginate) (Figs 36, 39, 42); (2) ~subtriangular or subrectangular, ~1.5 x long as wide (Figs 44, 46, 49, 52); (3) about as long as wide, sub-symmetrical (Figs 55, 58, 61, 64)

41. Intra-saccular process: (0) absent; (1) slightly raised bump, flange, or paddle centrally located on inner face of valva (Figs 36, 39, 42, 44, 46); (2) trigger-like extension at outer margin of lower lobe of valva (Figs 49, 52, 55, 58, 61, 64)

42. Intra-saccular process, adornment: (0) denticled or rugose (Figs 39, 44, 46, 49, 52, 55, 58, 61, 64); (1) naked (Figs 36, 42)

43. Saccular bend: (0) absent (Figs 36, 39, 42, 44); (1) present (Figs 46, 49, 52, 55, 58, 61, 64)

44. Saccular margin: (0) angled close to vinculum (Figs 46, 49, 52); (1) angled or rounded with apex at saccular midpoint (Figs 55, 58, 61, 64)

45. Saccular bend angled versus rounded: (0) angled, 90 degrees (Figs 61, 64); (1) rounded (Figs 55, 58)

46. Ventro-medial setal comb: (0) absent; (1) present (Figs 55, 58)

47. Localized patch or cluster of ventral, saccular spine-like setae: (0) absent; (1) present (Figs 36, 39, 42, 46, 49, 52, 55, 58, 61, 64)

48. Ventro-marginal setae: (0) absent or rudimentary (Figs 36, 39, 42, 44); (1) distributed along length of outer margin of sacculus (Figs 61, 64); (2) localized or concentrated at ventral bend (Figs 46, 49, 52, 55, 58)

49. Isolation of costal bar: (0) <75% along length of costa (Figs 46, 49, 52); (1) > 75% along length of costa (Figs 55, 58, 61, 64)

50. Secondary lobe of valva: (0) absent (Figs 46, 49, 52); (1) present, extending not beyond distal end of costa; (2) pronounced, finger-like (Figs 55, 58, 61, 64)

51. Recurved or decumbent setal plume associated with end of costa: (0) absent (Figs 36, 39, 42); (1) present (Figs 44, 46, 49, 52, 55, 58, 61, 64)

52. Setae arranged in recurved hook-shaped cluster: (0) absent; (1) present (Figs 58, 61, 64)

53. Scales arranged in terminal black dots on male abdomen: (0) absent; (1) present, conspicuous (all ingroup taxa)

54. Phallus - cornuti: (0) absent (Figs 37, 40, 43, 44); (1) present (Figs 47, 50, 53, 56, 59, 62, 65)

55. Number of cornuti: (0) one (Fig. 47); (1) two (Figs 50, 53, 56, 59, 62, 65)

**Female Genitalia [Figs 38-63, part]**

56. Antrum: (0) present, chalice-like (Figs 38, 41); (1) elongate, diffuse/indistinct from ductus bursae (Figs 45, 48, 51, 54, 57, 60, 63)

57. Colliculum: (0) absent (Fig. 38); (1) present, even if inconspicuous as in *chanesalis* (Figs 41, 45, 48, 51, 54, 57, 60, 63)

58. Narrow, differentially sclerotized band around center of colliculum: (0) absent (Figs 41, 45, 48, 54, 60); (1) present (Figs 51, 57, 63)

59. Ductus bursae: (0) effectively absent or inconspicuously short (Figs 38, 41); (1) present, variously sclerotized anterior to colliculum (Figs 45, 48, 51, 54, 57, 60, 63)

60. Sclerotization on floor of ductus bursae: (0) absent, indiscernable (Figs 45, 48); (1) present, either weak and diffuse or strong and conspicuous (Figs 51, 54, 57, 60, 63)

61. Corpus bursae: (0) elongate (Figs 38, 41, 45, 48); (1) globular (Figs 51, 54, 57, 60, 63)

62. Attachment of ductus bursae to corpus bursae: (0) basal (Figs 38, 41, 45); (1) sub-basal, creating shoulders on corpus bursae, associated with migration of point of attachment of ductus seminalis to sub-basal position of corpus bursae (Figs 48, 51, 54, 57, 60, 63)

63. Modification of Sternum 8: (0) absent; (1) present (all ingroup taxa)

After surveying all the described *Schacontia* species and determining there was insufficient evidence to retain *Schacontia replica* and *Schacontia pfeifferi*, these characters were scored (Appendix I) for three described *Schacontia* species (*medalba*, *chanesalis* and *ysticalis*), eight undescribed species, and three outgroups (*Eustixia pupula*, *Glaphyria sesquistrialis*, and *Hellula undalis*).

**Figures 21–29.**
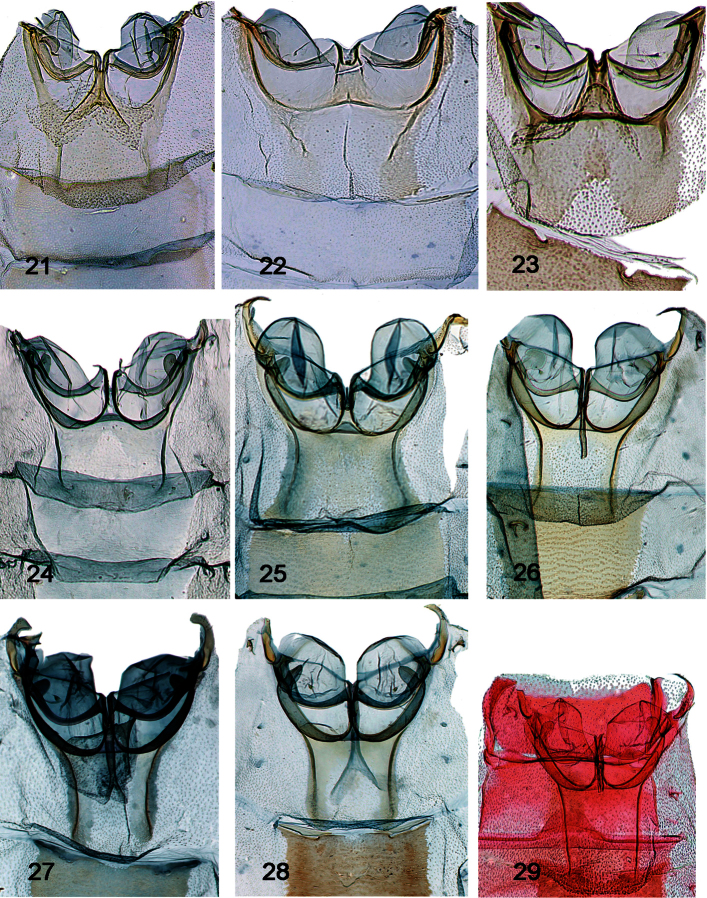
Tympanal organs. Collection and/or dissection numbers follow country of origin; when label data presented elsewhere, annotated as such. **21** *Schacontia medalba*, Brazil, “Bnito Prov., Pernmbuco, Brazil 83; Collection C.V. Riley; USNM genitalia slide 107887 **22** *Schacontia chanesalis* male, Guatemala: Quirigua Guat; Schaus and Barnes coll; Genitalia slide by DA ♂ USNM 107,891 **23** *Schacontia umbra* male Holotype, Ecuador, Past. Mera: 1300 m, xii.1992, V.O. Becker Col; Col. Becker 100504 **24** *Schacontia speciosa* male, Paratype, Brazil (data Fig. 4) **25** *Schacontia ysticalis* male 108100; Venezuela Guarico, Huato Masaguaral , 45 km S Calabozo, 8.57°N, 67.58°W, Galry For#4 75 m, 13–16 May 1988, uv light, M. Epstein R. Blahnik **26** *Schacontia themis*, male, Costa Rica, Guanacaste, Santa Rosa Nat’l Pk., 97 SRNP 2354.1 JAL, 2 May 2003, #5 **27** *Schacontia rasa* male Holotype, Mexico, Col. Becker 110514, Mexico, Tam San Fernando, 50 m, 28.vi.1997, V. O. Becker Col., [green USNM genitalia slide label “JAL 18”] **28** *Schacontia nyx* male, Venezuela, Lara, 4 km NW of La Pastora, 2–3.III.1978, riparian forest, blacklight, J.B. Heppner, Genitalia slide by DA ♂ USNM 108,101 **29** *Schacontia clotho* Cf Fig. 9.

**Figures 30–35.**
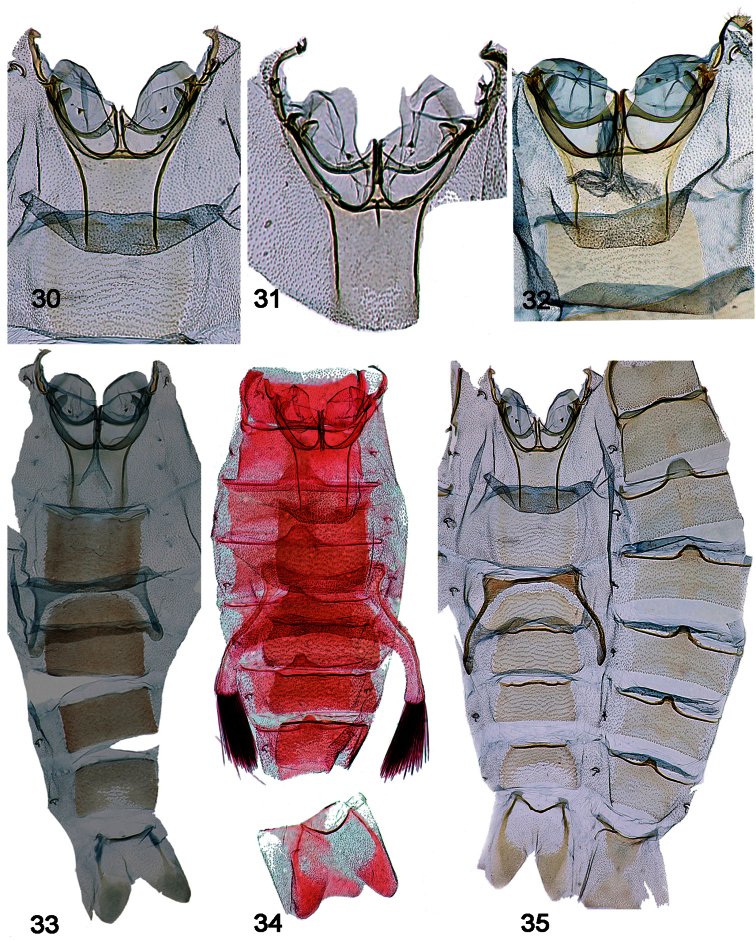
Tympanal organs and male abdominal segments. **30**–**32.** Tympanal organs. **30** *Schacontia lachesis* male, Bolivia, Cf. Fig. 11 **31** *Schacontia lachesis* female, Bolivia, Cf. Fig. 11 **32** *Schacontia atropos* male, Venezuela, Cf. Fig. 12 **33**–**35.** Male abdominal segments, illustrating coremata. **33** *Schacontia nyx* Venezuela, Cf. Fig. 22 **34** *Schacontia clotho* holotype, Catamayo, Ecuador, 1287, Cf. Fig. 9 **35** *Schacontia lachesis* Bolivia, Cf. Fig. 24.

**Figures 36–43.**
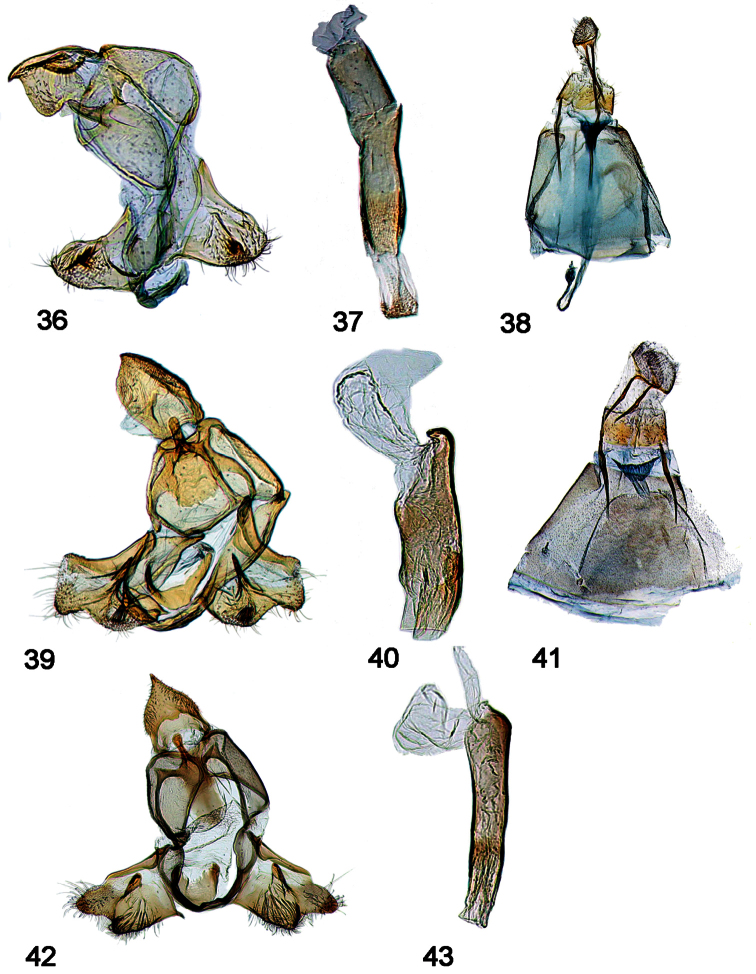
Male, female genitalia. **36** *Schacontia medalba* male, Brazil 107887 **37** *Schacontia medalba* phallus, data as above **38** *Schacontia medalba* female, Brazil, Castro, Parana, Collection Wm Schaus, Genitalia slide by MAS ♀ USNM 107,011 **39** *Schacontia chanesalis* male, Guatemala USNM 107,891m, Cf. Fig. 22 **40** *Schacontia chanesalis* phallus, data as above **41** *Schacontia chanesalis* female, Guatemala, Quirigua Guat., Schaus and Barnes coll, Genitalia slide by DA ♀ USNM 107,892 **42** *Schacontia umbra* male, Ecuador VOB 100504, Cf. Fig. 3 **43** *Schacontia umbra* phallus, data as above.

**Figures 44–51.**
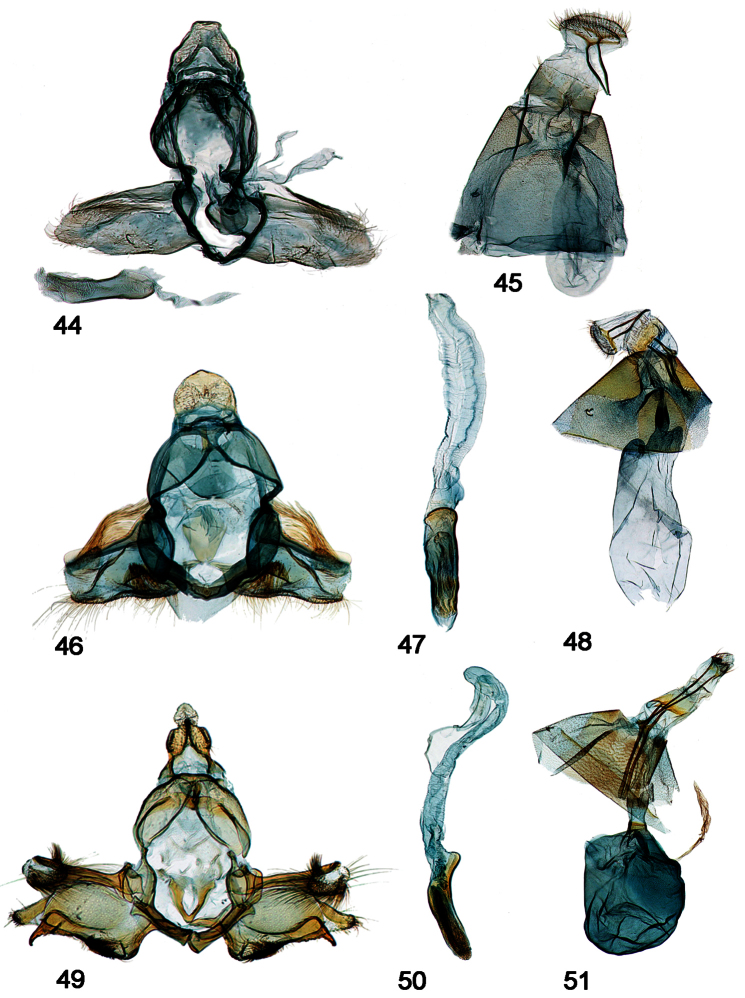
Male, female genitalia. **44** *Schacontia speciosa* male, paratype, Brasil, VOB 65271, Cf. Fig. 4 zzz**45** Female, Brasil: BA Jequié, 600–750m; Col. Becker 105714 **46** *Schacontia ysticalis* (a) male, Venezuela: Guarico, Huato Masaguaral , 45km S Calabozo 8.57°N, 67.58°W, Galry For#4 75m, 13-16May1988, uv light M. Epstein R. Blahnik; green label Genitalic Slide by DA ♂ USNM 108,100 **47** Phallus, data as above **48** Female, Venezuela Guarico, Huato Masaguaral , 45km S Calabozo Slide by DA ♀ USNM 107,896 **49** *Schacontia themis* (a) male, Costa Rica, Cf. Fig. 20 **50** Phallus, data as above **51** Female, Costa Rica, Guanacaste: Santa Rosa Nat’l Pk. 97 SRNP 2354.2 JAL 2 May 2003 #6.

**Figures 52–60.**
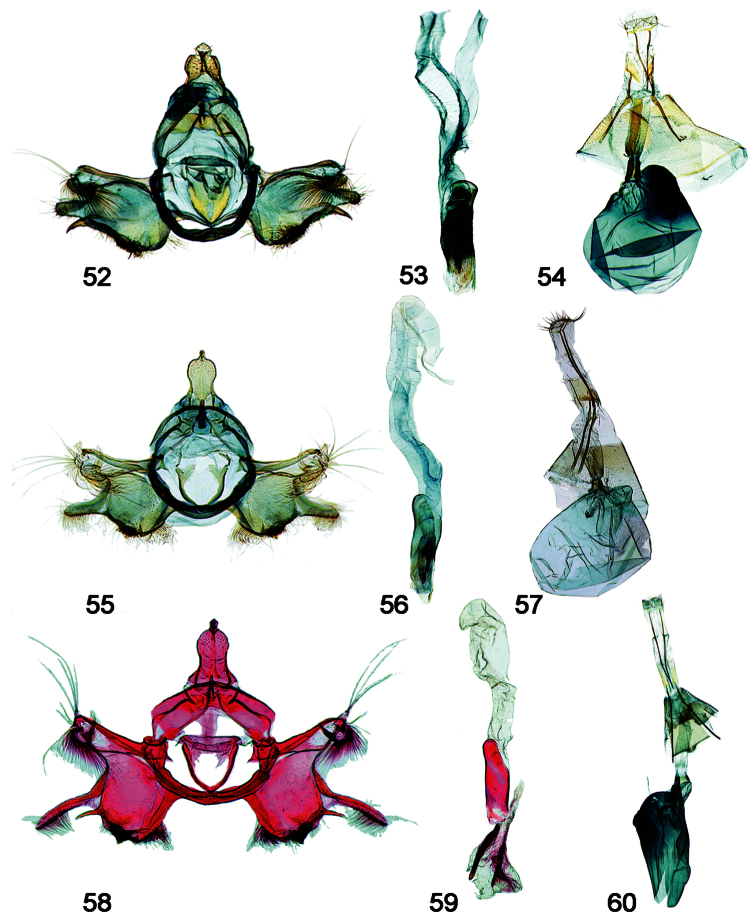
Male, female genitalia. **52** *Schacontia rasa* (a) male, Mexico; f. Fig. 21 **53** Phallus, data as above **54** Female, Mexico JAL 19 (same data) **55** *Schacontia nyx* (a) male, Venezuela, Cf. Fig. 22 **56** Phallus, data as above **57** Female, Venezuela; Cf. Fig. 8 **58** *Schacontia clotho* (a) male, HOLOTYPE, Ecuador; Cf. Fig. 9 **59** Phallus, data as above **60** Female, Ecuador: Loja, Catamayo, 1300 m, 20 XII 1992, V.O. Becker JAL 5 May 2003 #24.

**Figures 61–63.**
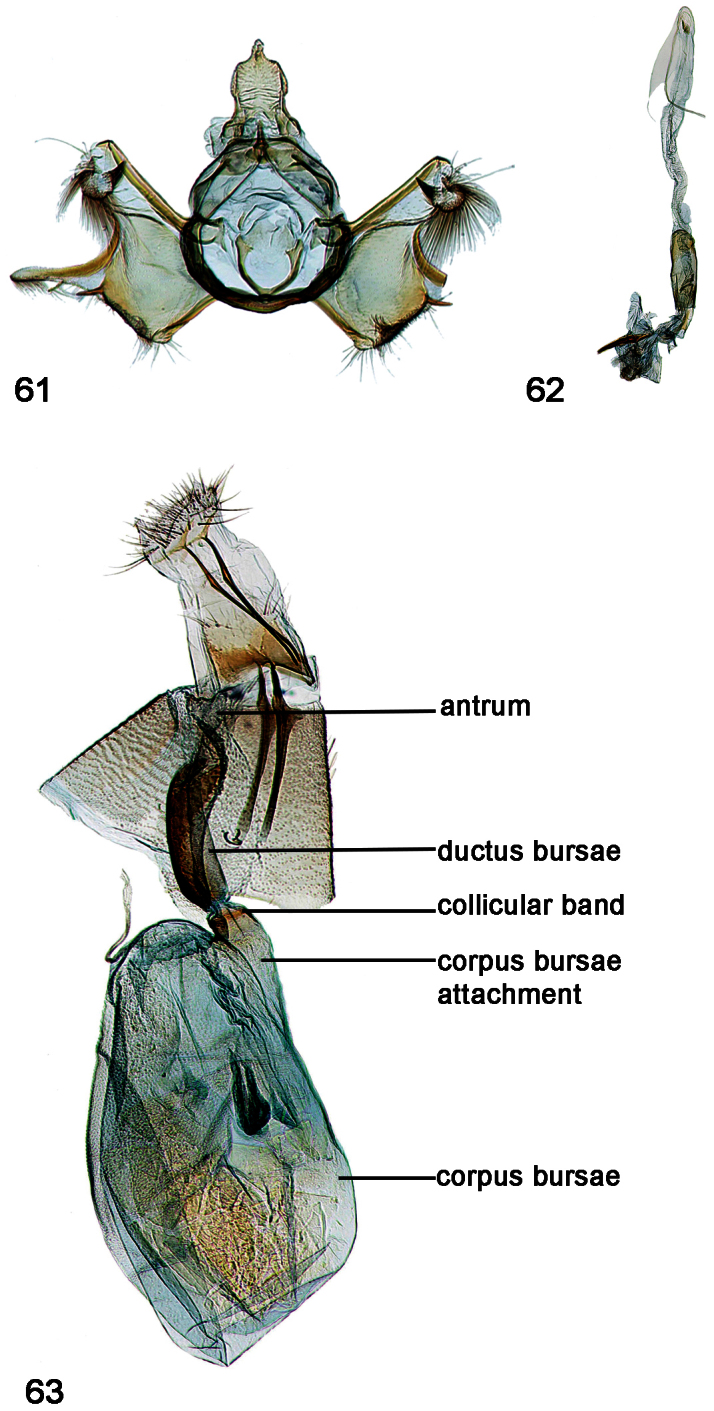
Male, female genitalia. **61** *Schacontia lachesis* (a) male, HOLOTYPE, Bolivia, CMNH; Cf. Fig. 11 **62** Phallus, data as above **63** Female, Brasil: MT 60 km S Pocone. Pantanal 100m 1-7.xii. 1997; V.O. Becker Col.; Col Becker 111257.

**Figures 64–65.**
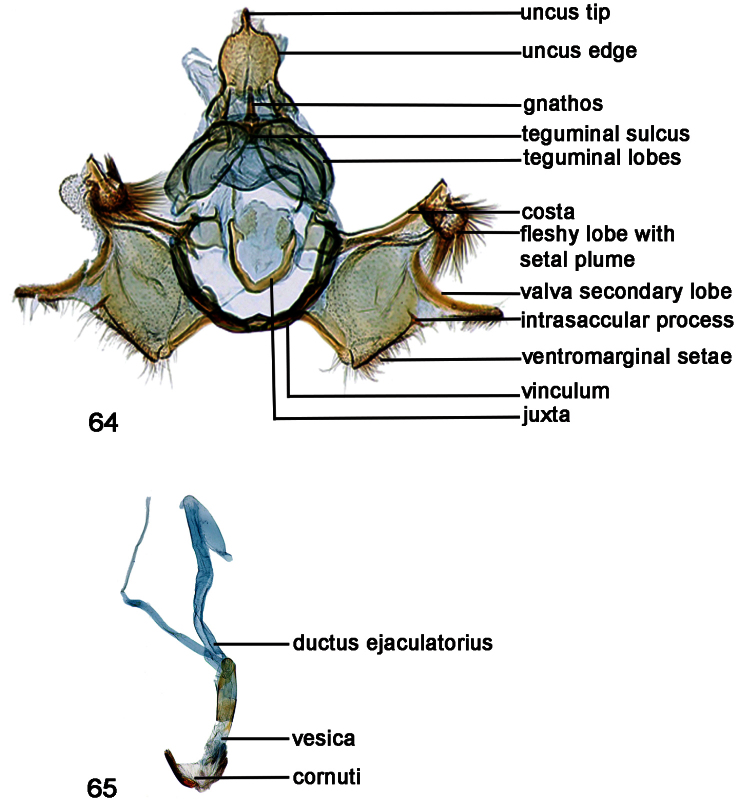
**64** *Schacontia atropos*, male, Venezuela; Cf. Fig. 12 **65** Phallus, data as above.

### Phylogenetic relationships

We were unable to discern consistently different characters among *Schacontia chanesalis*, *Schacontia pfeifferi*, and *Schacontia replica*, but in view of there being extremely limited material of *Schacontia pfeifferi* in particular, and despite Amsel’s description’s being the only detailed and well-figured one to date, we elected to synonymize *Schacontia replica* and *Schacontia pfeifferi* with *Schacontia chanesalis* based in large part on a lack of discernable discrete variation in the male genitalia.

**Figure 66.**
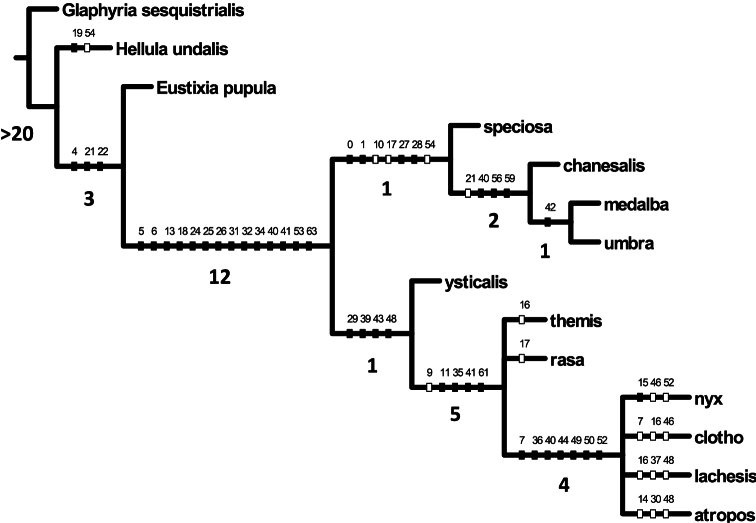
Strict consensus (L=108, CI=67, RI=81) of eight most parsimonious trees (L=102, CI=71, RI=84) obtained from a cladistic analysis of morphological data with unambiguous character state transformations indicated. Numbered hatch marks on nodes refer to characters undergoing forward changes (solid=unreversed; hollow=reversed). Bremer values are indicated below relevant branches.

From cladistic analysis eight most parsimonious trees obtain (L=102, CI=71, RI=84), the strict consensus of which (L=108, CI=67, RI=81) is presented (Fig. 66) with the topology: *Glaphyria sesquistrialis* [root] + [*Hellula undalis* + [*Eustixia pupula* + [[*Schacontia speciosa* + [*Schacontia chanesalis* + [*Schacontia medalba* + *Schacontia umbra*]]] + [*Schacontia ysticalis* + [*Schacontia rasa* + *Schacontia themis* + [*Schacontia lachesis* + *Schacontia atropos* + *Schacontia nyx* + *Schacontia clotho*]]]]]].

The monophyly of *Schacontia* is supported by synapomorphies enumerated in the diagnosis of the genus (below). Two primary groupings appear in the strict consensus (character numbers given parenthetically). The first comprises the type *Schacontia medalba*, *Schacontia chanesalis* and the newly described species *Schacontia umbra* and *Schacontia speciosa*; these are united by the absence of ocelli (0); reduced proboscis (1); a compound, non-uniform ground color that does not sharply delineate the medial area (8); a distinct hindwing postmedial line approaching or reaching the inner margin (10); robust, broad fornix (27); and wide, gently tapered venulae secundae (28). In these species the outer margin of the valva is also continuous, the valva highly reduced in all but *Schacontia speciosa* (40), and without a conspicuous saccular bend; phallus simple, without cornuti (55); attachment of the ductus bursae basal (63). Three of these species (excluding *Schacontia speciosa*) are characterized by having the saccus tympani deep, with a posterior ridge, but not invaginated posteriad (19) and a tipped, mucronate uncus that is not conspicuously obovoid and is longer than its width at the base. As will be discussed, 4 of 5 described species - all but one of which fall within this group - were described on the basis of female types. The morphology of the female genitalia is rather uniform among the species in this group; their putative association with males is based on a combination of wing pattern and geographical proximity.

The second major intrageneric grouping, the *ysticalis*-*themis* group comprises *Schacontia ysticalis* and six newly described species, whose association with *Schacontia* had been hypothesized initially. This group is united by a forewing pattern that is either essentially unicolorous excepting the antemedial and postmedial lines and orbicular spot, or two toned, but with the basal area unbroken and the medial area contrasting with the basal and apical areas (8); an inconspicuous hindwing postmedial line not nearing the inner margin (10); the saccus tympani a capacious ovate chamber with a conspicuous broad lip, comparable to that of *Cybalomia* Lederer (19); the dorsal ridge of the tegumen cruciate, crossing near the base of the uncus (33); the uncus either gently tapered and bluntly rounded, wider at the base than long, or variously nippled, trefoiled, and/or conspicuously obovoid (35, 36); the outer margin of the valva with upper and lower lobes, not with a continuous edge (39), but with a saccular bend or elbow either at its midpoint or proximal to the vinculum (44, 45); ventro-marginal setae present and well-developed (48); costal setae present, sometimes arranged in a recurved, fish-hook-shaped cluster (52); phallus with cornuti (54); attachment of the ductus bursae sub-basally, creating the shouldered appearance on the corpus bursae (62).

Morphologically, this second, perhaps more enigmatic species-group, is less homogeneous than that surrounding the type species of *Schacontia*. Its most basal member (*Schacontia ysticalis*) retains numerous features common to the latter group, viz. concentration of white scales apical to the postmedial line (character 9), the narrow distance between the postmedial line and the wing terminus (character 11), the light wing lines in contrast with the dark ground color (character 12), the undifferentiated uncus (character 35), the configuration of the intra-saccular process (character 41), and the elongate corpus bursae (character 61). The remaining species, all hitherto undescribed, form a complex of species exhibiting a heterogeneous collection of male secondary sexual characters, including unusual metatarsal structures, tibial scales and spurs, and abdominal coremata. These appear somewhat homoplastically, such that their downweighting or removal results in a more decisively resolved topology, but we retain them in analysis to emphasize their relevance to future work.

#### Schacontia

Dyar, 1914: 400

http://species-id.net/wiki/Schacontia

##### Type species.

*Acontia medalba* Schaus, 1904: 163, by subsequent designation.

##### Type locality:

Brazil.

##### Etymology.

“*Schacontia*” seems to be Dyar’s contraction of Schaus and *Acontia*, the noctuid genus in which Schaus mistakenly attributed *medalba* and subsequently designated by Dyar as the type species of *Schacontia*.

##### Diagnosis.

*Schacontia* species may be recognized by (character numbers listed parenthetically): Foreweing Rs_3_ and Rs_4_ stalked (5); M_1_ and M_2_ stalked (6); hindwing M_2_M_3_ + CuA_1_ stalked (13); bullae tympani invaginated in S2 (18); absence of puteoli (22); fornix heavily sclerotized and far removed from the edge of Ve1 (24); fornical angle a low arc > 90 degrees (25); presence of gnathos-ventrotergal rods complex (31), bearing a finger-like middle process (32); presence of teguminal sulcus (34); intrasaccular process a bump or flange towards base of valve or as a trigger-like process at margin of lower lobe of valve (41); pair of terminal black dots on abdominal dorsum of male (53); uncus hood-like, mucronate, or obovoid, with variously modified terminal nipple (35, 36). In addition, the costal bulge in the FW postmedial line is frequently coupled with a color contrast between the FW medial area and the basal and terminal areas, often involving white scaling. Unlike the *medalba* group (for present purposes including *Schacontia speciosa*), the proboscis is not reduced in the *ysticalis-themis* group, the labial palpi droop, the tympanal fornix is narrow, ribbonlike; venulae secundae tapered to form a “neck.”

##### Habitus.

In the species most readily identifiable as *Schacontia* (by virtue of their similarity to the type species *Schacontia medalba*), hereafter referred to as the *medalba* group, the forewings are gray with a metallic sheen and the antemedial and postmedial lines variously suffused with white, the exception being *Schacontia umbra*, which may be almost uniformly shaded dark brown. Towards the costa, the postmedial line bulges outward; the hind wings are by and large nondescript in pattern beyond the presence of a faint postmedial line. The *ysticalis*-*themis* group including the *Schacontia themis-rasa* sister pair and the *Schacontia nyx* complex [*Schacontia nyx*+*Schacontia clotho*+*Schacontia lachesis*+*Schacontia atropos*], are distinguished from these in having ocelli present; frons with normal, convex contour, except in *Schacontia ysticalis*; and labial palps porrect, extending beyond the clypeus.

##### Male genitalia.

All *Schacontia* bear a modification of the intrasaccular region of the valva. In the case of those species surrounding the type species of *Schacontia*, this comprises a naked or denticled flange; the valvae are characteristically reduced, if not truncate, and the uncus prominent but unadorned, mucronate. The valvae become progressively more complex in the *ysticalis*-*themis* group, with the intrasaccular feature transposed laterally to form a sclerotized trigger-like structure. Also in the *ysticalis*-*themis* group: the dorsal ridges of the tegumen are cruciate, meeting near the uncus; the tegumen is much wider than the uncus such that the lateral edges of the tegumen appear to taper/fall away from the uncus gradually; the outer margin of the valva is complex, including a variously adorned subcostal process, the costa associated with a fleshy lobe at its terminus and at least one setal tuft; the sacculus bears a localized patch or cluster of setae ventrad; and a membranous area exists between the costa and the subcostal process.

##### Description.

**Head** - In *medalba* group, ocelli and chaetosemata absent; proboscis reduced; frons conical; labial and maxillary palpi straight. In *ysticalis-themis* group, ocelli present; frons of normal, convex contour except in *Schacontia ysticalis*; labial palps porrect, extending beyond clypeus. **Thorax** - In *medalba* group, pronotum, mesonotum, legs gray; hind leg of female with 1 pair of tibial spurs. Males of several members of *ysticalis-themis* group bear a flattened, hind tibial spur, specialized hind tibial scales, a shallow concave spoon-like metatarsal modification, and coremata on 4^th^ abdominal segment (on *Schacontia themis*, *Schacontia nyx*, *Schacontia clotho*, *Schacontia lachesis*, and *Schacontia atropos*); in addition, epipleural setae may be present (in *Schacontia rasa*, *Schacontia clotho*, *Schacontia lachesis*, and *Schacontia atropos*); and female hind tibia usually bear two pair of spurs (a medial pair present) except in *Schacontia ysticalis* and *Schacontia rasa*. *Forewing* (FW) - *Schacontia* exhibit a characteristic curvature of postmedial line, outwardly bulging towards costa. In *medalba* group FW medial area partially suffused with white; in *ysticalis-themis* group, FW either unicolorous with basal and postmedial areas or polymorphic, with some specimens more darkly shaded. Rs_3_ and Rs_4_ stalked; M_1_ and M_2_ stalked. *Hindwing* - In *medalba* group, HW generally pale with few contrasting markings; female frenulum with a single seta; postmedial line sometimes present, conspicuous, but never in *ysticalis-themis* group. [M_2_M_3_]+CuA_1_ stalked. **Abdomen** - Scales arranged in two terminal black dorsal spots in males, more conspicuous in *ysticalis-themis* group. Tergites gray with dark-gray scaling in *medalba* group. Tympanal organs crambiform (tympanum and conjunctivum not co-planar, praecinctorium present, bullae tympani open anteromedially), but somewhat variable. In *medalba* group, bullae tympani broad, tympanal assemblage wider than long (cf. Solis 2009: 503); processi tympani present, towards antero-lateral end of fornix, prominent, lamellate, hemi-circular; processus spiniformis present; fornix tympani strongly sclerotized, broad, removed from edge of venula prima; fornical ulna gradually arched at approximately >90° angle; pons short, broad, V-shaped, length more or less equivalent to breadth of fornix; rami (posteromedial margins of sacci) weakly sclerotized, arcuate, not strongly angled medially; venulae secundae present, tapering gently such that posterior width only slightly less than anterior width; puteoli absent; posterior lip of saccus weakly sclerotized, saccus indistinct and grading into second sternite; posterior width of tympanal organs narrower than anterior width, but venulae secundae not tapering sharply to form a neck; bullae not conspicuously invaginated in S2. In *ysticalis-themis* group, tympanal assemblage less asymmetrical than in *medalba* group (i.e., not conspicuously wider than long); tergo-sternal sclerite robust, conspicuous; bullae tympani longer than wide, saccus or rim of bullae tympani sclerotized at base; processi tympani present, lamellate, thumb-like, towards antero-lateral end of fornix; fornix tympani sclerotized; angle of fornical ulna obtuse; pons elongate, comprising (in part) two parallel, elongate, sclerotized prongs, divergent only at anterior terminus (posteromedial margin of saccus appears delimited by sclerotized rami, extends and remains parallel to pons for most of its length, pons extending towards bottom of saccus); saccus deep, pronounced (cf. “poches ou dépressions tympaniques” of Minet 1985); venulae secundae prominent, tapering such that “partie libre” (*sensu* Minet) of second sternite forms a “neck” as in *Schacontia speciosa*; puteoli absent; posterior width of tympanal organs roughly half of anterior width. **Male genitalia** (Figs 36–60, part). *Medalba* group: Uncus oblong, cuspidate or mucronate, terminal edge entire; tegumen robust, divided into two dihedral, di-trapezohedral, or hemispherical bubbles that meet for a length that varies across species such that its dorsal ridges appear cruciate; juncture may appear as an elongate strut that divides anterior to base of uncus, such that anterior margin of tegumen may appear moderately emarginate (as in *Schacontia chanesalis*) or more deeply invaginate (as in *Schacontia medalba* and *Schacontia umbra*). A transparent, membranous or sub-sclerotized area within uncus overlies a finger-like process arising from within center of gnathos, configuration harness-like, comprising a plate suspended by four arms, one pair extending to and (apparently) articulating with base of uncus dorso-caudally; other subtergal pair extending ventrally to and articulating with vinculum; connection between gnathal plate and tegumen membranous. Lower arms of gnathos appear to represent a fusion with ventro-tergal rods (Cf. Yoshiyasu 1985). Characteristic reduced male valvae extend straight out at roughly a 90° angle, and with a localized patch or cluster of ventral, filiform saccular setae. Valvae either simple and rounded or broadly emarginate to bilobed; reduced, their most prominent feature a pair of intra-saccular processes (one in each valva) oriented dorsally and variously naked or adorned with spines or denticles. Ventro-marginal setae absent or rudimentary. Juxta U-shaped or broadly V-shaped, robust at base, vaguely taurean. Phallus simple, cornuti absent. *Ysticalis-themis* group: Uncus obovoid or superficially tridentate (appearing trefoil- or spade tipped); tegumen robust, divided into two obliquely-oriented oval sections meeting caudally near base of uncus, but diverging widely cephalad such that anterior margin of tegumen appears deeply invaginated; gnathos comprising a suspended rectangular plate with arms arising from each corner and a small, nub-like process arising centrally; dorsal arms wrap around anal tube, a ventral pair extend to termini of vinculum, such that gnathos almost appears to articulate both with uncus-tegumen and with vinculum, which is variously U-shaped or horseshoe shaped with pronounced pockets at each terminus. Valvae complex, comprising regions and processes that are variously sclerotized, fleshy in appearance, and/or bearing tufts of setae: intrasaccular flange located towards latero-ventral edge and sclerotized to form a trigger-shaped process; robust, spine-like setae on valva; ventro-marginal setae present on valva, either distributed evenly along length of outer margin of sacculus or concentrated at ventro-saccular “ulna”; costa robust and joined to rest of valva by a narrow membranous area; valva with secondary outer fleshy setose lobe or process below costa; recurved/decumbent setal plume associated with terminus of costa. Juxta robust, V-shaped or broadly U-shaped, ventral tip curved outward forming a small chin-like platform in *Schacontia themis* and *Schacontia rasa*; a less robust, more open U-shape in *Schacontia nyx* complex. Phallus with two cornuti. **Female genitalia** (Figs 38–63, part) - *Medalba* group: Papillae anales convex, partially appressed but separate, setose; posterior and anterior apophyses roughly equivalent in length, not especially robust; antrum may be conspicuous, chalice-like; ductus bursae short, not discretely circumscribed; corpus bursae membranous, elongate, without signa; ductus seminalis arising from posterior end of corpus bursae. *Ysticalis-themis* group: Papillae anales setose, rounded, not conspicuously dihedral (except in *Schacontia lachesis*); colliculum, if present, a partial collar, sometimes shortened to form a narrow ring immediately outside corpus bursae, ductus bursae per se all but eliminated; note that in contrast to *Udea* Guenée (1845), for example, ductus bursae, if present, developed posterior to colliculum (cf. Mally and Nuss 2011: 63, fig. 3), an elongate band or partial sleeve immediately occupying antrum, appearing as a sclerotized band on floor of ductus bursae; corpus bursae globular or ovoid (more elongate in *Schacontia ysticalis*), without signa, one or two accessory bursae posteriad where ductus seminalis attached.

**Species variation.** Individual species variation with respect to wing polymorphism is especially acute in the *Schacontia nyx* complex; of particular interest here are the male secondary sexual characteristics, which covary imperfectly across species and are discussed below. *Schacontia* species may vary greatly in size (>100% wingspan).

**Distribution.** Collectively, *Schacontia* species are distributed across Mexico, south to Central America (Guatemala, Costa Rica, Panama) and South America (Bolivia, Brazil, Ecuador, Venezuela) and the Caribbean (Puerto Rico, Cuba, Hispaniola). A single North American record of *Schacontia themis* is reported here from Sanibel Island, Florida (USA: Lee Co.).

##### Biology.

Larvae are internal feeders that may induce galls, and pupate within the host. The only known host plant records are in Capparaceae: in Costa Rica, larvae have been reared from *Podangrogyne decipiens* (Triana & Planch.) Woodson (Solis, Nishida and Metz, in preparation); *Cleome spinosa* Jacq. has been reported as host for *Schacontia chanesalis*; *Capparis frondosa* Jacq., and *Capparis verrucosa* Jacq. are reported for other *Schacontia* species.

##### Remarks.

*Schacontia* was described by Dyar (1914) to accommodate three species, whose original descriptions were based primarily on wing pattern: the type species *Schacontia medalba* (Schaus, 1904; formerly *Acontia medlba*); *Schacontia chanesalis* (Druce, 1899), formerly *Pionea chanesalis*; and *Schacontia replica* Dyar, 1914, the last of which accompanied the generic description (Druce 1899: 557, Schaus 1904: 163, Dyar 1914: 400). Dyar (1925: 8) later described *Thlecteria ysticalis* from a female specimen, also on the basis of wing pattern, and this species was later removed to *Schacontia* by Munroe (1995: 42), who also recognized *Schacontia pfeifferi* Amsel, 1956, raising the total number of species recognized in the genus to five. Amsel’s (1956: 101–102) description of *Schacontia pfeifferi*, which placed *Schacontia* in the Schoenobiinae, is the most complete description to date and one of only two works prior to the present to figure or characterize genitalia (the other being Solis 2009). Neither Schaus nor subsequent authors were explicit in their characterization of what makes *Schacontia* unique or in their rationale for describing and including new species in the genus.

##### Key to species of *Schacontia*

Key to species of *Schacontia*: Male Genitalia + Habitus + Female genitalia (part)

**Table:** 

1	Forewing generally silvery gray or gray brown with white shading in vicinity of antemedial and postmedial lines, particularly in medial area and at outermost edge of postmedial line; or dark brown with poorly contrasted markings except for postmedial line. Hindwing postmedial line conspicuous, nearing inner margin. Frons conical. Valva simple or reduced, sub-quadrate or emarginate/mildly bilobed; lacking a straight, prominent coastal arm; medial projection or flange arising from within sacculus; apex of costa lacking a tuft or plume, or a fleshy, setose subcostal lobe. Uncus mucronate, hoodlike. Tegumen dividedwherein the two tablet-shaped, bubble-like sections meet centrally for some or most of their length. Juxta more or less horseshoe shaped. Tympanal apparatus with saccus indistinct, posterior ridge lightly if at all sclerotized, grading into second sternite; venulae secundae not sharply tapering inward caudally; fornix broad, robust	2
1’	Forewing ground color straw or light gray, uniform or with a contrasting gray medial area suffused with white; or with outer margin and basal areas rust colored (*Schacontia ysticalis*) or yellowish brown (*Schacontia lachesis* and *Schacontia atropos*); antemedial and postmedial lines conspicuous; HW postmedial line faint, without secondary postmedial shading, not reaching inner margin. Frons with normal undifferentiated convex contour. Valva broad with a distinct costal bar or boom and either a faint, rudimentary hump appearing in the ventro-saccular region or as a more prominent, lateral sclerotized process at ventro-marginal edge of sacculus; a tuft or plume associated with costal terminus, each of which may also bear a fleshy, setose subcostal lobe. Uncus, either gently tapering to a wide rounded tip, or obovoid or squared, bearing a trefoil-like tip, in the last case appearing nearly tridentate, with a raised central ridge resulting in a webbed appearance (this feature may vary in prominence). Tegumen deeply divided such that two ovular sections meet obliquely towards base of uncus. Juxta robust, V- or broadly V-shaped. Tympanal saccus distinct, with posterior ridge or lip heavily sclerotized; venulae secundae tapered inward caudally; fornix narrow, ribbon-like.	4
2(1)	Forewing medial area variously but diffusely shaded, generally without sharp contrast or orbicular spot; basal area not traversed by a white band. Uncus tapering to a distinct, ventrally directed squared tip. Tegumen invaginate such that sulcus joining two teguminal hemispheres extends less than 40% of length of tegumen. Intra-saccular process smooth, not conspicuously denticled	3
2’	Forewing medial area variously shaded, but often with contrast, an orbicular spot varyingly distinct or inconspicuous, if present; the basal area usually traversed by a white band. Uncus broadly tapered with a simple rounded nipple. Teguminal sulcus extends most of length of tegumen. Intra-saccular process rugose or denticled	*Schacontia chanesalis*
3(2)	Forewing color variously shaded with white scaling; lines or variously shaded regions conspicuous. Orbicular spot faint, if present. Hind wing slate gray. Valva truncate, rounded, entire	*Schacontia medalba*
3’	Forewing shaded chocolate brown, markings not obvious. Hind wings dark gray. Valva slightly emarginate	*Schacontia umbra*
4(1’)	FW medial area suffused with white basad; postmedial line with broad, gentle costal bulge. Uncus dorsoventrally flattened, edges nearly carinate; uncus tip broad, neither acutely sharp nor sculpted with trefoil shape. Valva lacking a trigger-like process below costa; costa with mane-like tuft of elongate setae, recurved medially. Phallus simple, cornuti absent. Corpus bursae elongate, without signa	5
4’	FW shading either unicolorous or with medial area more darkly shaded than both basal and postmedial areas. Uncus tip swollen, either obovate or squared, in latter case with lateral edges thickened. Valva with a trigger-like process arising from within sacculus along ventral edge, and a conspicuous fleshy subcostal lobe and setose plume; costa lacking tuft of elongate setae medially recurved. Phallus with two prominent cornuti. Corpus bursae more or less globular, rarely with signa	6
5(4)	Basal area of FW with a brown ovoid spot, delineated by white bands crossing from wing base to antemedial line. Uncus tapered towards blunt squarish tip at a roughly 60 degree angle. Valva entire, not emarginate, without distinct upper and lower extensions; center of valva unadorned; intra-saccular structures indistinct. Phallus simple, naked, without cornuti	*Schacontia speciosa*
5’	Basal area of FW rust colored, mottled. Uncus broad, lateral edges parallel, tapering to a wide, gently rounded tip at a roughly 45 degree angle. Valva with upper and lower extensions, the lower sclerotized dorsad; intrasaccular flange conspicuous, adorned with both surficial and adjacent setal clusters. Phallus with a single cornutus.	*Schacontia ysticalis*
6(4’)	FW uniform mouse gray or mottled, in latter case with medial area more darkly shaded. Uncus rounded, obovate with distinct, rhomboid nipple; uncal edges not reinforced or swollen. Valva gently rounded ventrally with moderate to elongate lateral process, ventro-medial edge with a distinct comb of elongate setae	7
6’	FW ground color gray or straw colored, contrasting gray medial area in some specimens. Uncus squared or scooplike in appearance with lateral edges swollen, sometimes conspicuously so, with or without a pronounced central ridge, tip hastate or trefoil-like; valva either elbowed or sharply angled towards mid-point, but not gently rounded, lacking an elongate process distally, ventro-medial edge without a distinct comb of elongate setae	8
7(6)	FW mottled in appearance, medial area slightly darker than basal and postmedial areas; orbicular spot pronounced. Epipleural setae absent. Ventral trigger-like process on valva rudimentary, if present; subcostal lobe robust, squat, <=3x longer than wide	*Schacontia nyx*
7’	FW gray, unicolorous; orbicular spot faint. Epipleural setae present. Ventral trigger-like process pronounced; subcostal lobe elongate and narrow, ~5x longer than wide	*Schacontia clotho*
8 (6’)	Uncus with conspicuous, prominent central ridge. Elongate lateral lobe of valva absent; subcostal lobe not elongate; ventral edge of valva not conspicuously elbowed close to vinculum; central membranous area of valva conspicuously longer than wide	9
8’	Uncus with a uniformly smooth contour. Subcostal lobe pronounced, finger-like; ventral edge of valva angled or elbowed sharply (not rounded) approximately mid-way between vinculum and lateral edge of valva; central membranous area of valva not conspicuously longer than wide	10
9(8)	Female with two pairs of hind tibial spurs; male with coremata present on 4^th^ abdominal segment, flattened hind tibial spur, specialized hind tibial scales with embedded dark patch, cuplike metatarsal modification, epipleural setae	*Schacontia themis*
9’	Female with single pair of hind tibial spurs; male secondary sexual features above absent	*Schacontia rasa*
10(8’)	Male secondary sexual characters (including coremata on 4^th^ abdominal segment, cf. 10) all present	*Schacontia lachesis*
10’	Male secondary sexual characters (cf. 10) absent	*Schacontia atropos*

#### Schacontia medalba

(Schaus, 1904)

http://species-id.net/wiki/Schacontia_medalba

Figs 1 , 21 , 36–38

Acontia ? *medalba* Schaus, 1904: 163. Type Locality: Brazil.

##### Material examined.

(19♂, 10♀, 1 sex undet.).

##### Type material.

Holotype (♀, USNM): Castro, Parana; Collection Wm Schaus; [red type label] type 10575; Acontia? medalbi [sic] sp. Schs; Pyralie Schoenobiana gen. nov.; USNM Genitalia Slide by DA ♀ 107,899.

##### Other material examined.

**Brazil:** (19♂, 8♀, 1 sex undet.): Bnito Prov. Pernmbuco Brazl 83, unknown [illegible] 84 W.S. 165, genitalia slide by DA ♂ USNM 107,909 (1♂); Bnito Prov., Pernmbuco Brazl 83, [illeg.], Collection C.V. Riley, Genitalia slide by DA ♂ USNM 107,887 (1♂); Bnito Prov., Pernmbuco Brazl 83, [illeg.], Collection C.V. Riley, Genitalia slide by DA ♀ USNM 107,888 (1♀); Pernambuco, Brazil, coll. Pickel, 17 II 929 2065, Genitalia slide by DA ♀ 107,910 (1♀); Pernambuco Tapera, 1934.VIII.24, 2087♀ (1♀); Bnito Prov., Pernmbuco, Brazl. 6/1 83, Not known [illeg.], Collection of C.V. Riley ♂ (1♂); Pernambuco, Brazil, Coll. Pickel ♂ (1♂); 28, Bnito Prov., Pernmbuco, Brazl 8 3, 2, Fernald ♂ (1♂); 73, Not in BM 1925, W Schaus ♂ (1♂); 2♂ 1♀, as previous; Castro, Parana, Collection Wm Schaus, incl. 1: Genitalia slide by MAS ♀ USNM 107,011 (1♀); 1♂ as previous; Brazil: Nova Teutonia, F. Plaumann (1♂); Col. Becker No. 4601, Rio Brilbante, Mato Grosso, Brasil, 25.I.1971, V. O. Becker Col., *Schacontia medalba* det. M.A. Solis [on one only] (2♀); Col. Becker No. 9164, Rio Brilbante, Mato Grosso, Brasil, 24.X.1970, Becker leg. (1♀); Nova Teutonia, 27°11’S, 52°23’W, Brazil, 300–500 m, 4-IV-1954, Fritz Plaumann [CNC] (4♂); Nova Teutonia, 27°11'S, 52°23'W, Brazil, 300–500 m, 10-IV-1954 Fritz Plaumann [CNC] (4♂); as above, “Slide No. 3645M.S.” (1 sex undet.); Nova Teutonia, 27°11'S, 52°23'W, Brazil, 300–500 m, 3-III-1954, Fritz Plaumann, *Schacontia* n. sp. 7, Det. E.G. Munroe 1998 [CNC], Genitalia slide by JAL ♂ (1♂). **Peru** (1♀): Boniti P, Peru, Jan 7. 83.

##### Diagnosis.

Specimens of *Schacontia medalba* are most readily diagnosed from those of *Schacontia chanesalis* by male genitalia, specifically the reduced, unlobed valvae and the naked intrasaccular process, features they share with *Schacontia umbra*.

##### Re-description

(Fig. 1). Forewing length 6.5–1.0 mm. **Head** *-* Frons conical; labial palpi straight, extending as far as clypeus. **Thorax** - Female with one pair of hind tibial spurs (medial pair absent); legs uniform gray brown. *Forewing*. Basal area primarily gray brown, undivided; antemedial (am) line meets anal margin. Subterminal line interrupted by wing veins; medial area partially suffused with white, especially basad; white postmedial line appears shaded basally, interrupts/traverses dark shading between apical area and distal region of medial area; this “double” line faintly common to HW; FW fringe gray-brown.* Hindwing*. Postmedial line present, conspicuous (see above); terminal area lightly shaded, fringe white. **Abdomen** *-* Apical bands of pale scales on abdominal segments; terminal dots grayish brown, faint if present. *Tympanal organs* (Fig. 21). As for the *medalba* group, *vide supra*. **Male genitalia** (Figs 36, 37). Teguminal sulcus short, such that anterior margin of tegumen appears deeply invaginate; juxta U-shaped; valvae simple, reduced, rounded, not bilobed or emarginate; intrasaccular process a simple flange; intrasaccular process naked; phallus simple, cornuti absent. **Female genitalia** (Fig. 38). Antrum wider than deep, chalice-like; ductus bursae inconspicuous, no colliculum apparent; corpus bursae indistinct, weakly sclerotized, elongate.

##### Immature stages.

Unknown.

**Variation.** Variable in size; forewings vary with respect to obfuscation in medial area.

##### Biology.

Unknown.

##### Distribution.

Brazil, Peru.

#### Schacontia chanesalis

(Druce, 1899)

http://species-id.net/wiki/Schacontia_chanesalis

Figs 2 , 13 , 15–17 , 22 , 39 , 41

Pionea chanesalis Druce, 1899, p. 557.
Schacontia replica Dyar, 1914, p. 400, **syn. n.** (Holotype ♀, Mexico, USNM).
Schacontia pfeifferi Amsel, 1956, p.101, **syn. n.** (Holotype ♂, Guatemala, ZSM, Munich).

##### Material examined.

Below we summarize material examined for *Schacontia chanesalis*. We include material previously determined as its new synonyms, *Schacontia replica* and *Schacontia pfeifferi*, and list them accordingly. We acknowledge that cryptic species may yet be identified pending the accumulation of molecular data.

##### Type material.

Holotype (♀, BMNH): Holotype [round white label w/ red border]; El Tumbador, Guatemala, Champion; Godman-Salvin, Coll. 1904-1., B.C.A. Lep.-Het., Pionea chanesalis Druce; Pionea chanesalis Druce, type [hand written]; Genitalia Slide by DA, ♀. [Holotype of *Schacontia replica*]: March 1912, Orizaba, Mex, 3414, R Muller Collector, [red type label] Type 15484, Schacontia replica Dyar Type, [green label] USNM Genitalia Slide by DA ♀ 107,898, left FW missing (1♀). [Holotype; ZSM, Munich]; Typus ♀ leg. H. Amsel; Venezuela Maracay leg. P. Vogl.; Genitalia slide by DA 108,040 ♀, det. Amsel 1953 Schacontia pfeifferi Ams. [“Allotype”, ZSM, Munich].

##### Other material examined.

**Costa Rica** (8♂, 11♀, 4 sex undet.): Santa Rosa National Park Guanacaste Prov. Costa Rica 2–4 May 1980 DH Janzen & W. Hallwachs, Genitalia Slide by DA ♂ USNM 107,903, INBio Barcode # CR1001 115186 (1♂) as previous [no slide label], #CR1001 115190 (1♀); Estac. Quebrada Bonita, 50m R.B. Carara Puntarenas Pr. Costa Rica Nov 1989. R. Zuniga. 194500, 469850, Genitalia Slide by DA ♂ USNM 105,819, head illustrated; INBio Barcode # CR1000 120043 (1♂); Estac. Quebrada Bonita, 50m R.B. Carara Puntarenas Pr. Costa Rica April 1989. R. Zuniga. 194500, 469850, Genitalia Slide by DA ♂ USNM 106,418 [v. poor specimen], INBio Barcode # CR1001 103073 (1♂); Estac. Quebrada Bonita, 50m R.B. Carara Puntarenas Pr. Costa Rica Oct 1989. R. Zuniga. 194500, 469850, Genitalia Slide by DA ♀USNM 105,820, INBio Barcode # CR1000 160925 (1♀); Estac. Quebrada Bonita, 50m R.B. Carara Puntarenas Pr. Costa Rica Set 1989. R. Zuniga. 194500, 469850, INBio Barcode # CR1001 103076 (1♀[?]); Est. Sta. Rosa, 800m, P.N. Guanacaste, Prov. Guan. Costa Rica, I. Curso Microlepidopt., Jul 1990 L-N-313000, 359800, INBio Barcode # CR1000 182323 (1♀); Est. Maritza, 600 m, Lado oeste del Volcan Orosi I curso Microlepidopt., July 1990 L-N-326900, 373000, INBio Barcode # CR1000 181312 (1♂); Santa Rosa National Park Guanacaste Prov. Costa Rica 7–9 July 1980 DH Janzen & W. Hallwachs, INBio Barcode # CR1001 115188 (1♀); Santa Rosa Nat. Pk., Prov Guanacaste, Costa Rica 10–12 Nov 1979 D.H. Janzen, INBio Barcode # CR1001 115187 (1♂); 97-SRNP-320, 8, Genitalia Slide by JAL ♀ (1♀); 97-SRNP-320, [right FW detached] (1♀); Prov. Guanacaste, Z.P. Nosara, Sector of Mirador, 800 m 2–8 Nov 2002. H. Mendez. Tp. De Laz. L N 220750 383450 #72175, INB0003554509 (1♀); Estac. Quebrada Bonita 50m R.B. Carara Puntarenas Pr. Costa Rica Oct 1989. R. Zuniga. 194500, 469850, INBio Barcode # CR1000 196823 (1♂); Fca. Cafrosa, Est. Las Mellizas, P.N. Amistad, 1300m Prov. Punt. COSTA RICA M. Ramirez & G. Mora, Nov. 1990 L-S-316100-596100, INBio Barcode # CR1000 278769 (1♀); Estac. Quebrada Bonita, 50m R.B. Carara Puntarenas Pr. Costa Rica Set 1989. R. Zuniga. 194500, 4698500 (1 sex undet.); Est. Sta. Rosa, 800m, P.N. Guanacaste, Prov. Guan. Costa Rica, I. Carso Microlepidopt., Jul 1990 L-N-313000, 359800 (1 sex undet.); Schacontia sp. Crambidae, Costa Rica, Cartago prov. Parque National Tapanti near the ranger station 1250m, 25-V-2005 (adult emergence) Col/rear: Kenji NISHIDA Host Plant: Podandrogyne decipiens (Capparidaceae), gall inducer on the stem unknown family, female (1♀); Schacontia sp. Crambidae, Costa Rica, Cartago prov. Parque National Tapanti near the ranger station 1250m, 25-V-2005 (adult emergence) Col/rear: Kenji NISHIDA Host Plant: Podandrogyne decipiens (Capparidaceae), gall inducer on the stem, Schacontia n. sp. 2/06 det. M.A. Solis (1 sex undet.); Schacontia sp. Crambidae, Costa Rica, Cartago prov. Parque National Tapanti near the ranger station 1250m, 25-V-2005 (adult emergence) Col/rear: Kenji NISHIDA Host Plant: Podandrogyne decipiens (Capparidaceae), gall inducer on the stem Deformed adult caught in its pupal shell x1 Pupated 15-VI-2005 (pupal stage 1 month) (1♂); Schacontia sp. Crambidae, Costa Rica, Cartago prov. Parque National Tapanti near the ranger station 1250m, 25-V-2005 (adult emergence) Col/rear: Kenji NISHIDA Host Plant: Podandrogyne decipiens (Capparidaceae), gall inducer on the stem unknown family, female (1♀); Schacontia sp. Crambidae, Costa Rica, Cartago prov. Parque National Tapanti near the ranger station, 1250m, 25-V-2005 (adult emergence) Col/rear: Kenji NISHIDA Host Plant: Podandrogyne decipiens (Capparidaceae), gall inducer on the stem unknown family, male (1♂); Costa Rica: Estac. Biol. Las Cruces 6 km SE San Vito Rio Jaba 1150m X-20/21/1993, blacklight in secondary forest J. Powell coll. (1sex undet.); Voucher: D.H. Janzen & W. Hallwachs DB: http//Janzen.sas.upenn.edu Area de Conservacion Guanacaste Costa Rica 97-SRNP-320.1, “legs away for DNA” (1 sex undet.); same as previous, 97-SRNP-320.2, 97-SRNP-320.3, and 11-SRNP-12677 (1♂,1♀, 1 sex undet., respectively). **Guatemala:** Cayuga Guat, Dec, Schaus and Barnes coll, Genitalia slide by DA ♂ USNM 108,097 (1♂); Quirigua Guat, Schaus and Barnes coll, Genitalia slide by DA ♀ USNM 107, 892, FW in capsule (1♀); Grutas de San Pedro Martir, Guatemala Escuintla VIII-10-1965 P.J. Spangler 1♀[?]. **Honduras**: El Hatillo Honduras Black light 3-VIII-1972 Robert D. Lehman (1 sex undet., obscured by mold). **Mexico** (22♂, 3♀, 3 sex undet.): Col. Becker 44006, Mexico: Veracruz Huatusco 1300m 19–23. Viii. 1981 V.O. Becker col., Comp. c/tipo USNM 1981 V.O. Becker (1♂); Nov ’11, Orizaba Mex, R Muller collector, 3414, Chanesalis or [illeg.] desc. as Pionea [illeg.] Schoenobiinae, ♀ USNM 197,890 Genitalia slide by DA (1♀); Mexico: 2 mi. N. Tamazunchale, S.L.P. 400’, July 16–18, 1963, Duckworth & Davis, Genitalia slide by DA ♂ USNM 108,099 (1♂); Mexico: 2 mi. N. Tamazunchale, S.L.P. 400’, July 16–18 1963, Duckworth & Davis, Genitalia slide by DA ♂ USNM 108,889 (1♂); Mexico: El Salto Falls, 26 mi W. Antiguo Morelos, Tamps., 2000’, July 11–14 1963, Duckworth & Davis (1♀); Mexico: .2 mi. N. Tamazunchale, S.L.P., 400’, Aug. 2 1963, Duckworth & Davis (1♂); (17♂, 1♀, 1 sex undet.[genitalic slide unavailable, “1291”]): Col. Becker 68741, Mexico, Tam El Ensino, 250 m, 4–13.viii.1988, V.O.Becker Col., Genitalia Slide ♂ by JAL [one specimen only]; Col. Becker 108733, Mexico: Tam El Encino, 250 m, 21–31.v. 1997, V. O. Becker Col. (1♂); Mex: Ver.,7 km NNW Huatusco, 1300 m, VIII-15-1987, J.T. Doyen (1 sex undet.). **Venezuela** (3♂): Venezuela: Guarico, Hato Masaguaral, 45 km S Calabozo, 8.57N, 67.58W, Galry Forest #20, 75 m, 3–5 June 1988, uv light, M. Epstein Genitalia slide ♂ by JAL USNM 108,083] (1♂); VENEZUELA: San Esteban Carabobo, Venez., Dec. 1–20 1939, Pablo J. Anduse, Illustration of wing pattern (1♂); Venezuela Maracay leg. P. Vogl., Jan.–Febr.35, Typus ♂ leg. H. Amsel, Genitalia slide by DA 108,039 ♂ (1♂).

##### Diagnosis.

Specimens of *Schacontia chanesalis* are best distinguished from those of *Schacontia medalba* by the male genitalia, specifically a more sinuate valva and more denticled or rugose (as opposed to naked) intrasaccular process. The valvae are less conspicuously lobate than in *Schacontia umbra* (below). Forewing pattern somewhat variable, as in *Schacontia medalba*, but antemedial area more often traversed by white bar originating at scapula, enhancing the baso-costal patch.

##### Re-description

(Fig. 2). Forewing length: 4.5–9.0 mm. **Head** *-* Ocelli and chaetosemata absent; proboscis reduced. Labial palpi porrect, extending slightly beyond clypeus. Frons conical; vertex and frons grayish brown, intermixed with white scales medially and along anterior bases of antennae. **Thorax** *-* Prothoracic collar light gray intermixed with gray-brown and white scales. Tegula and mesoscutum mostly gray, intermixed with light-gray and/or grayish-brown scales, the posterior apex of tegulae pale gray. Legs predominantly white, gray shading throughout foreleg; female with one pair of hind tibial spurs (medial pair absent). *Forewing*. Baso-costal triangle flanked by white scaling towards inner margin and in medial area, which is outwardly shaded brown (suffused with white basad). Postmedial area (between postmedial line and subterminal line) grayish brown. Subterminal line white; terminal line black, interrupted. Marginal scales brown. Basal area grayish brown traversed by a white band. Fringe scales light gray. *Hindwing*. Ground color white/very light gray, darker postmedially; postmedial lines grayish brown, white distally, conspicuous and common with FW. Subterminal area shaded darker brown; fringe white. Sc+R1 and Rs anastomosed slightly beyond dilated base of former. Male and female acanthae of frenulum fused from near base to apex to form one bristle. **Abdomen** *-* Ground color mostly dark gray intermixed with gray and light-gray scales above, white on undersurface. *Tympanal organs* (Fig. 22). As above for *medalba* group, *vide supra*. **Male Genitalia** (Figs 39, 40) *-* Tegumen divided dorsally into two dihedral or hemi-spherical “bubbles” that meet at a central sulcus, which divides anterior to base of uncus and forms a Y-shaped strut. Teguminal sulcus long, extending length of two teguminal lobes, such that anterior margin of tegumen appears emarginate, but not deeply invaginated. Uncus oblong, mucronate or miter-like, culminating in a distinct tip; concave or spatulate, setose on inner (ventral) surface. A membranous, more or less circular region at base of uncus positioned directly above (dorsal to) finger-like projection of gnathos, which also comprises a floating sub-tegumenal plate with four arms. Finger-like process arises from center of gnathos; dorsal pair of arms, which meet at juncture of uncus and tegumen, appearing to fulfill traditional description of gnathos by enveloping the anal tube, and the anterior pair extending ventrolaterally towards the vinculum, resembling a wishbone. Gnathos thus appears as a subtegumental (ventrad) suspension. Valvae reduced, broadly emarginate, bilobed; intrasaccular process a simple flange, denticled or rugose; subapical setal cluster near saccular margin. Costa robust, curved, appearing to arise near the respective vincular terminus. Juxta horseshoe-shaped, the base wider than the lateral “arms.” Phallus simple, moderately sclerotized throughout; cornuti absent. **Female Genitalia** (Fig. 41) *-* Papillae anales appressed; antrum apparent, chalice-like; ductus bursae short; corpus bursae elongate, without signa, caeca, or appendix bursae; ductus seminalis originating from posterior portion of corpus bursae. Ostium bursae with membrane between seventh and eighth segments.

##### Biology.

Larvae have been reared from *Podandrogyne decipiense* (Capparaceae) (D. Janzen, pers. comm.).

##### Distribution.

Mexico, Guatemala; Costa Rica; Venezuela.

##### Immature stages.

Unknown.

##### Variation.

In size, with Mexican specimens appearing smaller in wingspan (FW length 4.5–7.0 mm) than Central American specimens.

##### Remarks.

It is with some trepidation that we synonymize both *replica* and especially *pfeifferi* with *chanesalis*. *Pfeifferi* in particular was, until this work, the only *Schacontia* for which a detailed description had been published, and its continental separation from the type locality and primary distribution of *chanesalis* might suggest the potential for as yet unrecognized diagnosable species.

#### Schacontia umbra

Solis & Goldstein sp. n.

urn:lsid:zoobank.org:act:524F3C52-1E37-4E82-B0F8-AF4485B49E2C

http://species-id.net/wiki/Schacontia_umbra

Figs 3 , 23 , 42, 43

##### Material examined.

**Type material. Ecuador**: Holotype **(**♂, USNM): Ecuador, Past. Mera: 1300 m xii. 1992 V.O. Becker Col; Col. Becker 100503, (1♂). Paratypes (4♂), USNM. Ecuador, Past. Mera: 1300 m xii. 1992 V.O. Becker Col, Col. Becker 100503, (♂); Ibidem, 100504, (1♂); Ecuador, Past. Puyo: (22 km W) 5 February 1976 Blacklite [sic] Spangler, et al., Ecuador, Peace Corps. Smithsonian Institution Aquatic Insect Survey, Genitalic slide by DA ♂108,095, (1♂); (27 km N) Est. Fluv. Metrica 4 February 1976 Spangler, et al. (1♂).

##### Diagnosis.

*Habitus, male genitalia* (Figs 3, 42). This species is most readily diagnosed by the darkly shaded forewings and by the male genitalia, which have the following features in common with *medalba*: anterior margin of tegumen deeply invaginate, outer margin of valva entire, intra-saccular process naked.

##### Description.

(Fig. 3). Forewing length: 7.5–8.0mm (n=5). **Head** - Ocelli absent; proboscis reduced; frons conical; labial and maxillary palpi straight, not extending beyond clypeus. **Thorax** - Female with one pair of hind tibial spurs (medial pair absent). *Forewing*. Shaded gray brown, hind wings dark gray brown; postmedial line, when evident, characteristic of genus, outwardly bulged towards costa, sinuous towards inner margin, but entire forewing more darkly shaded than in other species. Medial area partially suffused with white in some but not all specimens. Subterminal line pale, unbroken; fringe gray. *Hindwing*. Uniformly brown gray; subterminal line pale tawny, unbroken; fringe gray. **Abdomen** *-* Uniformly covered in gray-brown scales. *Tympanal organs*. (Fig. 23). As for *medalba* group, *vide supra*. **Male genitalia** (Figs 42, 43) *-* Teguminal sulcus short, such that anterior margin of tegumen appears deeply invaginate; juxta a U-shaped plate; valvae simple, reduced, rounded, not bilobed or emarginate; intrasaccular process a simple flange, naked; phallus simple, cornuti absent. **Female genitalia** (Fig. 44) *-* Unknown.

##### Immature stages.

Unknown.

##### Variation.

Markings may be obscured in some specimens, rendering them more or less uniformly gray brown.

##### Etymology.

The specific epithet refers to the dark wing shading of this species and is treated as a noun in apposition.

##### Biology.

Unknown.

##### Distribution.

Central Ecuador.

#### Schacontia speciosa

Solis & Goldstein sp. n.

urn:lsid:zoobank.org:act:77C2883E-34CB-4725-8AEC-A6C530C487F9

http://species-id.net/wiki/Schacontia_speciosa

Figs 4 , 24 , 44, 45

##### Material examined.

**Type material. Brazil**: Holotypemale, USNM (Fig. 4): Col. Becker 65271; Brasil: RJ Marica 5m,11. x.1985, V.O.Becker Col. **Paratypes** 10♂, 1♀, 2 sex undet., USNM. Brazil: Same data as holotype (9♂, with additional label “Genitalia 1290”); BRAZIL: BA Jequié, 600–750 m; Col. Becker 105714 (1♀); BRAZIL: Rio Jan. 10 km SW Maricá “restinga” sand dune, 11–12-X-85 Scott E. Miller (1♂, 2 sex undet, ex abd.).

##### Diagnosis.

*Habitus, male genitalia*(Figs 4, 44). The forewing pattern of this species makes it unmistakable; readily diagnosed by a combination of the frosted medial area common to other *Schacontia* and the interruption of the brown basal area to render a medio-basal patch encircled in white. Male genitalia diagnosed from those of other *Schacontia* species by the combination of expanded (not truncate) but inornate valvae, and reduced features associated with them, such as the inconspicuous intrasaccular flange; and a blunt, squarish, barely-tapering uncus.

##### Description.

Male (Fig. 4). Forewing length: 7.5–8.0 mm, (n=14). **Head** - Ocelli absent; proboscis normal; frons expressed as a small hump, but not conspicuously conical; labial palpi porrect, extending beyond clypeus. **Thorax** - Vertical scales mocha; female with one pair of hind tibial spurs (medial pair absent). *Forewing*. Medial area gray, partially suffused with white basad; postmedial line shaded white outwardly, brown inwardly; basal and submarginal areas primarily mocha brown; basal area interrupted by oblong basal patch surrounded by white. Subterminal line dark, unbroken; fringe scale gray, darkest at termini. *Hindwing*. Brownish white, no contrasting markings, postmedial line inconspicuous if present; subterminal line dark, unbroken; fringe scales brown, pale gray at margin. **Abdomen** *-* Scales arranged in two terminal black dorsal spots in males. *Tympanal organs* (Fig. 24). Tergo-sternal sclerite robust, conspicuous; bullae tympani longer than wide, saccus or rim of bullae sclerotized at base; processi tympani present, lamellate, thumblike, towards anterolateral end of fornix; processus spiniformis present; fornix tympani sclerotized; angle of fornical ulna obtuse; pons of intermediate length, roughly half the depth of saccus, component rods broad, separate along entire length, diverging at anterior termini; posteromedial margin of saccus extends and remains parallel to pons for most of its length, pons extending towards bottom of saccus; saccus pronounced; venulae secundae prominent, tapering slightly at base of tympanal case such that “partie libre” (*sensu* Minet) of second sternite forms a neck; puteoli absent. **Male genitalia** (Fig. 44) *-* Teguminal sulcus short, not apparent; juxta U-shaped, lateral arms modesty recurved; valvae simple, broad, not truncate; intra-saccular process rudimentary, if present; costa with recurved, elongate tufts of setae but no conspicuous fleshy lobe; phallus simple, cornuti absent. **Female genitalia** (Fig. 45) *-* Papillae anales separate, round, swollen; colliculum present as faintly sclerotized collar embedded in ductus bursae, which is short, inconspicuously delimited and unsclerotized anterior to colliculum; corpus bursae elongate, membranus, without signa or appendix bursae; ductus seminalis inserted between antrum and ductus bursae.

##### Immature stages.

Unknown.

##### Etymology.

The specific epithet is from the Latin for showy or handsome.

##### Biology.

Unknown. Adults active in October.

##### Distribution.

Southeastern Brazil (Bahia, Rio de Janeiro).

#### Schacontia ysticalis

(Dyar, 1925)

http://species-id.net/wiki/Schacontia_ysticalis

Figs 5 , 25 , 46–48

Thlecteria ysticalis Dyar, 1925, p. 8.

##### Material examined.

(16♂, 16♀, 1 sex undet.).

##### Type material.

**Mexico:** Holotype (♀, USNM).

##### Other mFaterial examined.

**Bolivia**: Puerto Suarez, Bolivia, 150 m, Dec. 1908, J. Steinbach C.M. Acc. 3758 (1♂) [CMNH]. **Costa Rica** (4♂, 6♀):Santa Rosa National Park, Guanacaste Prov., Costa Rica, D.H. Janzen, 12 Dec 1978–10 Jan 1979, Genitalia Slide by DA ♂ USNM 107,905, INBio Barcode # CR 1001 115170 (1♂); Santa Rosa National Park, Guanacaste Prov., Costa Rica, D.H. Janzen, 12 Dec 1978–10 Jan 1979, Genitalia Slide by DA ♂ USNM 107,904, INBio Barcode # CR 1001 115169 (1♂); Playa Naranjo, Sta Rosa P.N., Guanacaste Prov., Costa Rica, E. Alcazar, Ene 1991 L-N-309300-353300, INBio Barcode # CR 1000 640648 (1♂); Santa Rosa National Park, Guanacaste Prov., Costa Rica, D.H. Janzen, 12 Dec 1978–10 Jan 1979, Genitalia Slide by DA ♀ USNM 107,906, INBio Barcode # CR 1001 115171 (1♀); Est. Queb. Bonita, 50 m, Res. Biol. Carara, Prov. Punt., Costa Rica, R. Zuniga, Jun 1991, L-N-194500, 469850, INBio Barcode # CR 1000 343579 (1♀); Estac. Quebrada Bonita, 50 m, R.B. Carara, Puntarenas Pr., Costa Rica, R. Zuniga, April 1989, 194500, 469850 INBio Barcode # CR 1000 017910 (1♀); Sirena, Corcovado Nat. Pk., Osa Penin., Costa Rica, 19–27 Mar1981, DH Janzen, W. Hallwachs, INBio Barcode # CR 1001 115172 (1♀); Est. Quebrada Bonita, R.B. Carara, Prov. Punta, Costa Rica, 50 m, Mar 1994, R. Guzman, L N 194500_469850 #2803, INBio Barcode # CR1001 754072 (1♀); Est. Quebrada Bonita, R.B. Carara, Prov. Punta, Costa Rica, 100 m, ENE 1995, R. Guzman, L_N_195250_469850 #4433, INBio Barcode # CR1002 243527 (1♀); Costa Rica, Prov. Limon, Sector Cedrales de la Rita, 3 km N del Puente Rio Suerte, Ruta Puerto Lindo, 10 m, Feb 1997, E. Rojas, L_N_278600_566500 #45311, INBio Barcode # CR1002 499299 (1♂). **Mexico** (2♂, 4♀):Col. Becker 42358, Mexico: Veracruz Est. Biol. Tuxtlas, 11–16.vi.1981, V. O. Becker Col., *Schacontia ysticalis* Dyar, Det. M.A. Solis (1♀); Ibid. x4 exc. Col. Becker 42297 (3♀, 1♂); Venadio, Sinaloa, Mex, B P Clark donor, Not in BM 1925 W Schaus (1♂). **Nicaragua** [CMNH]: Managua Dist., Laguna de Xiloa, 23 April 1996, E. van den Berghe (3♂, 1♀); Managua Dist., Laguna de Xiloa, 14 April 1996, E. van den Berghe (2♂);Managua Dist., Laguna de Xiloa, 8 March 1997, E. van den Berghe (1♂) [CMNH]. **Venezuela** (7♂, 5♀): Venezuela, Guarico, Huato Masaguaral, 45 km S Calabozo, 8.57°N, 67.58°W, Galry For#4, 75 m, 12–13 Apr 1988, uv light, M. Epstein, R. Blahnik, Genitalic Slide by DA ♀ USNM 107,896 (1♀); same as previous (2♂, 4♀, 1 sex undet.), “hind leg used for illustration” [Fig. 20]); Venezuela, Guarico, Huato Masaguaral , 45 km S Calabozo, 8.57°N, 67.58°W, Galry For#4, 75 m, 13–16 May 1988, uv light, M. Epstein, R. Blahnik [3♂, incl. 1w/ label Genitalic Slide by DA ♂ USNM 108,100]; Venezuela, Guarico, Huato Masaguaral, 45 km S Calabozo, 8.57°N, 67.58°W, Galry For#4, 75 m, 23–24 Apr 1988, uv light, M. Epstein, R. Blahnik, Genitalic Slide by DA ♂ USNM 107,895, “Head illustrated” (1♂); Venezuela, Guarico, Huato Masaguaral, 45 km S Calabozo, 8.57°N, 67.58°W, Galry For#4, 75 m, 25 May, uv light M. Epstein & C. Canaday (1♂).

##### Diagnosis.

*Habitus, male and female genitalia* (Figs 5, 46–48). Distinct by virtue of orange cast to basal and postmedial areas of forewing. Male genitalia distinct by virtue of wide, rounded uncus without modifications; heavy, long recurved setal tufts at costa of valva; and intrasaccular patch of heavy setae. Female genitalia distinct by virtue of elongate, robust bursa combined with conspicuous appendices bursae.

##### Re-description.

Male (Fig. 5). Forewing length: 7.0–11.0 mm (n=14). **Head** (Fig. 14) - Ocelli present; proboscis normal; frons conical or expressed as a small hump; labial palpi porrect, extending beyond clypeus. **Thorax** - Legs white, forelegs cupreous dorsally, as basal tarsomeres on all legs. Female with two pair of hind tibial spurs (medial pair present). *Forewing*. Basal, antemedial, and subterminal fasciae brownish orange, shaded distally with white (dark basad). Both antemedial and postmedial lines shaded distally with white (dark basad); antemedial and postmedial areas rust colored/cupreous; medial area sparsely suffused with white. FW fringe brown. *Hindwing*. postmedial line faint if present; HW yellowed at margin, subterminal line interrupted; fringe whitish beige. **Abdomen** - Cupreous sheen; white abdominal bands on all segments. Scales arranged in two terminal black dorsal spots in males. *Tympanal organs* (Fig. 25). As for *ysticalis-themis* group, *vide supra*. **Male genitalia** (Figs 46, 47) - Teguminal sulcus short, such that anterior margin of tegumen appears deeply invaginate, the two oblong teguminal lobes joined obliquely. Uncus wider than long; terminal edge of uncus entire. Gnathos quadrate. Juxta an inverted triangular plate or robust “V”, less sclerotized at center. Valvae complex; costa robust with recurved, elongate tufts of setae; subcostal lobe with petiolate scales, most arched towards dorsal articulation of valva; with secondary outer, oblong lobe or process below costa; with fleshy setose lobe associated with terminus of costa and located between distal portion of costa and lower portion. Intrasaccular process a simple flange, the inner surface of which bears chisel-shaped setae; with robust, spine-like setae at base; submarginal area of sacculus setose. Saccular margin angled close to vinculum, not at saccular mid-point; ventro-marginal setae concentrated at saccular ulna. Phallus moderately sclerotized; vesica with a small cornutus. **Female genitalia** (Fig. 48) - Papillae anales separate, more or less round, flat, and swollen; ostium bursae with membrane between seventh and eighth segment; antrum membranous; colliculum present as a sclerotized collar of intermediate length embedded within ductus bursae; two conspicuous appendices bursae located at posterior end of corpus bursae; corpus elongate, membranous, without signa; ductus seminalis near posterior end of corpus bursae.

##### Immature stages.

Unknown.

##### Variation.

Unremarkable.

##### Biology.

Unknown. Recorded adult activity mid-June (Mexico), January–June (Costa Rica), 8 March–23 April (Nicaragua), 12 April–25 May (Venezuela), December (Bolivia).

##### Distribution.

Mexico, Costa Rica, Nicaragua, Venezuela, Bolivia.

#### Schacontia themis

Solis & Goldstein sp. n.

urn:lsid:zoobank.org:act:81B6D7E0-F6F0-44C8-B4B7-BB8424FE4B9F

http://species-id.net/wiki/Schacontia_themis

Figs 6 19, 26 49–51

##### Material examined.

**Type material.** Holotype (♂,CMNH). **Dominican Republic**: La Altagracia. 2 km N Bayahibe, 18-23N, 68-51W, 10 m, 3 July 1992, C. Young, R. Davidson, S. Thompson, J. Rawlins, Dry seasonal forest on limestone, USNM ENT 00808538, DNA 2012 (1♂) [GenBank Accession #KC789515]. Paratypes (41♂, 19♀) USNM, except where otherwise designated. Costa Rican paratypes with an INBio barcode label deposited at INBio. **Brazil** (5♂, 1♀, 1 sex undet.): Unit. Amaz. Taperinha b. Santarem 1–10 VI ’27, Zerny (1♂) [NHMV]; Unit. Amaz. Taperinha b. Santarem, 1–10 VIII ‘27, Zerny (1♂) [NHMV]; Unit. Amaz. Taperinha b. Santarem, 21–31 VII ’27, Zerny (1♂) [NHMV]; Unit. Amaz. Taperinha b Santarem 1–10 VII ’27, Zerny, 86 (1♂) [NHMV]; Col. Becker 105713, Brasil: BA Jequié, 600–750 m, 11–22 xi 1995, V.O. Becker Col. [1 sex undet.]; Unit. Amaz. Taperinha b. Santarem 1–7 IX ’27, Zerny, 43, genitalia slide by JAL ♀ USNM 108,870 (1♀); Unit. Amaz. Taperinha b. Santarem 1–10 VII ’27, Zerny, 43, Genitalia slide by JAL ♂ USNM 108,880 (1♂). **Cayman Islands** (7♂, 1♀): N.B. Certain Cayman Islands specimens have multiple data labels with conflicting dates. 17 iv–26.viii 1938, Oxf. Un. Cayman Is. Biol. Exped., Coll. by C.B., G.H. Thompson, 18 v 1938, Cayman Brac., N. coast of Stakes Bay, Light trap A, [yellow tag PARATYPE, *in errato*], *Proboscontia amica* Munroe, CNC (2♂); 17 iv –26 viii 1938, Oxf. Un., Cayman Is. Biol. Exped., Coll. by C.B. Lewis, G.H. Thompson, 4 v 1938, Grand Cayman, N coast of Rum Point, Light trap (1♂) [BMNH] [red Holotype label, *in errato*]; 17 iv –26 viii 1938, Oxf. Un. Cayman Is. Biol. Exped., Coll. by C.B. Lewis, G.H. Thompson, 1 vii 1938, Grand Cayman, East end of Interior, The Cliff, Light trap, ♀ Pyralidae Brit. Mus. Slide No. 19801, Slide No. 3663 MS (1♀) [BMNH]; 17 iv –26 viii 1938, Oxf. Un. Cayman Is. Biol. Exped., Coll. by C.B. Lewis, G.H. Thompson, 14 v 1938, Grand Cayman, East End light trap B (1♂) [BMNH]; 17 iv –26 viii 1938, Oxf. Un. Cayman Is. Biol. Exped., Coll. by C.B. Lewis, G.H. Thompson, 7 v 1938, Grand Cayman, N coast of, Rum Point, Light trap (1♂) [BMNH]; 17 iv –26 viii 1938, Oxf. Un. Cayman Is. Biol. Exped., Coll. by C.B. Lewis, G.H. Thompson, 22 v 1938, Cayman Brac., N coast of Stakes Bay, Light trap A, ♂ Pyralidae Brit. Mus. Slide No. 19802 (1♂) [BMNH]; 17 iv –26 viii 1938, Oxf. Un. Cayman Is. Biol. Exped., Coll. by C.B. Lewis, G.H. Thompson, 11 vii 1938, Grand Cayman, N coast of North Side, Light trap B (1♂) [BMNH]. **Costa Rica** (5♂, 6♀, 1 sex undet.): Guanacaste. Voucher INBio data base Costa Rica 97-SRNP-2346 Testigo Base de datos INBio Costa Rica (1♂); Voucher INBio data base Costa Rica 97-SRNP-2354.2 Testigo Base de datos INBio Costa Rica, Genitalia slide ♀ by JAL(1♀); Voucher INBio data base Costa Rica 97-SRNP-2349 Testigo Base de datos INBio Costa Rica (1♀); Sirena, Corcovado Nat. Pk. Osa Penin. Costa Rica 10–12 Aug. 1980, D.H. Janzen & W. Hallwachs, Genitalia slide by DA ♀ USNM 107,907, INBio Barcode #CR1001 115167, “Head illustrated” (1♀); Est. Cacao, 1000–1400 m, Lado SO Vol. Cacao, P.N.G. Prov. Guan, Costa Rica, C. Chaves, Abr 1991, L-N-323300, 375700 (1♀); INBio Barcode # CR1000 700522, Est. Santa Rosa, Prov. Guana, Costa Rica, 300 m, 25 Feb–5 MAR 1995, B. Gamboa, L N 313300 #4730 (1♀); INBio Barcode # CR1000 187409, Est. Sirena, 0–100 m, P.N. Corcovado, Prov. Punt., Costa Rica, G. Fonseca, May 1991, L-S-270500, 508800, INBio Barcode # CR1000 563282 (1♀); Voucher INBio data base Costa Rica 97-SRNP-2352 Testigo Base de datos INBio Costa Rica, “S. mootii #4” (1♂); Voucher INBio data base Costa Rica 97-SRNP-2354.1 Testigo Base de datos INBio Costa Rica, genitalia slide ♂ by JAL (1♂); Sirena, Corcovado Nat. Pk., Osa Penin., Costa Rica 5–11 Jan 1981, D.H. Janzen & W. Hallwachs, Genitalia slide by DA ♂ USNM 107, 908, INBio Barcode #CR1001 115168 (1♂); 80.SRNP.47, Santa Rosa National Park, Guanacaste Province, Costa Rica, D.H. Janzen, genitalia slide ♂ by JAL (1♂); 1sex undet.: Santa Rosa National Park, Guanacaste Prov., Costa Rica, 1 May 1980, D.J. Janzen & W. Hallwachs, INBio Barcode # CR1002 506841. **Cuba** (2♂):Col. Becker 72733, Cuba: Gtnmo Imias, 10 m, 17 vii 1990, V.O. Becker, 17, Genitalia slide by JAL ♂, USNM ENT 00808541, DNA 2012 (1♂); Col. Becker 73068, Cuba: Stgo. Siboney, 20 m, 23 vii 1990, V.O. Becker, 15, genitalia slide by JAL ♂, USNM ENT 00808540, DNA 2012 (1♂). **Dominican Republic** (1♂, CMNH): Same data as holotype, USNM ENT 00808539 [GenBank Accession # KC789516] (1♂). **Jamaica** (9♂, 1♀)**:** Jamaica: Clar. Par., Mason River Station 4 mi NW Kellits , DNA 2012, 2200 ft, 16–19 Apr ’73, Don & Mignon Davis (1♂); Jamaica: Clar. Par., Portland Ridge nr. Jackson Bay Cave, 40 ft, 4 May 1973, Don & Mignon Davis (1♂); Jamaica: Ann Par., nr. Runaway Bay Cave, 50 ft, 1–2 May 1973, Don & Mignon Davis (1♂); Jamaica: Clar. Par., nr. Jackson Bay Cave, 1.5 mi SE Jack. Beach, 50 ft, 4 May 1973, Don & Mignon Davis (6♂, 1♀) [incl. 1♂ genitalia slide ♂ by JAL USNM 108,879, + 1♀ genitalia slide ♀ by JAL USNM 108,868]. **Panama** (5♂, 2♀males): Panama: Canal Zone, Barro Colorado Isl., 21 Mar 1979, Silberglied/Aiello, at light, 35, Genitalia slide by JAL USNM 108,874 (1♂); Panama: Canal Zone, Barro Colorado Isl., 7 Mar 1979, Silberglied/Aiello, at light (1♂); Panama: Canal Zone, Barro Colorado Isl., 31 Mar 1979, Silberglied/Aiello, at light (1♂); Panama: Canal Zone, Barro Colorado Isl., 19 Mar 1979, Silberglied/Aiello, at light (1♂); Panama: Canal Zone, Barro Colorado Isl., 18 Mar 1979, Silberglied/Aiello, at light (1♂); Panama: Canal Zone, Barro Colorado Isl., 12 Mar 1979, Silberglied/Aiello, at light, 34, Genitalia slide ♀ by JAL USNM 108,873 (1♀); Panama: Canal Zone, Barro Colorado Isl.,1 Apr 1979,Silberglied/Aiello, at light (1♀). **Puerto Rico** (4♂, 2♀): Puerto Rico, Guanica, Bosque Estatal de Guanica, 3.6 km E Guanica, 17-58-11N, 66-52-28W, Thornscrub, 100 m, 12 June 1996, J. Rawlins, R. Davidson, C. Young, M. Klingler, W. Zanol, S. Thompson, Carnegie Museum Specimen Number 65,312 (1♂) [CMNH]; Puerto Rico, Guanica, Bosque Estatal de Guanica, 3.6 km E Guanica, 17-58-11N, 66-52-28W, thornscrub, 100 m, 12 June 1996, J. Rawlins, R. Davidson, C. Young, M. Klingler, W. Zanol, S. Thompson, Carnegie Museum Specimen Number 65,695 (1♂) [CMNH]; Col. Becker 67784, Puerto Rico, Guanica, 170 m, 20 viii 1987, V.O. Becker (2♂, 1♀ ) [incl. 1 genitalia slide ♂ by JAL]; Col. Becker 67782, Puerto Rico, Guanica, 170 m, 20 viii 1987, V.O. Becker (1♀). **Venezuela** (3♂):Venezuela, Guarico, Hato Masaguaral, 45 km S Calabozo 8.57N, 67.58W, Galry For #10, 75 m, 23–24 Apr 1988, uv lt., M. Epstein & R. Blahnik (2♂); Venezuela, Guarico, Hato Masaguaral, 45 km S Calabozo 8.57N, 67.58W, Galry Forest #20, 75 m, 13–16 May 1988, uv lt., M. Epstein & R. Blahnik [x2 incl. 1w/Genitalia slide ♂ by JAL USNM 108,869] (1♂) [Fig. 6].

##### Additional material examined:

**British Virgin Islands (BVI)** (29♂, 16♀): Brit. Virgin Isl., Guana Island, 0–80 m, 5–23 July 1985, S.E. & P.M. Miller, Clubhouse 60 m, U.V. light trap, 9–15 July 1985 (1♂); same as previous, 36, VOB ♀ USNM 108,875 (1♀); Brit. Virgin Isl., Guana Island, 0–80 m, 5–23 July 1985, S.E. & P.M. Miller (5♂); same as previous, VOB ♂ USNM 95900 (1♂); Col. Becker 66651**,** Brit. Virgin Isl., Guana I., 0–80 m, 9–23.vii.1987, V.O. Becker & S.E. Miller, Genitalia 1260 (1 sex undet.); Col. Becker 70821, Brit. Virgin Isl., Guana X. 1989 V.O. Becker (10♂, 3♀) [incl. ♂ “slide 21”, hair pencils and abdominal coremata present]; same as previous, Genitalia slide by JAL ♂ (1♂, 1♀); same as previous, Genitalia 1261 and 1262 (2♀); Col. Becker 66649, Brit. Virgin Isl., Guana I., 0–80 m, 9–23 vii 1987, V.O. Becker & S.E. Miller, 11, Genitalia Slide ♂ by JAL (1♂) [hair pencils and abdominal coremata present]; Col. Becker 66651, Brit. Virgin Isl., Guana I, 0–80 m, 9–23 vii 1987, V.O. Becker & S.E. Miller (1♂, 2♀); Col. Becker 66650, Brit. Virgin Isl., Guana I., 0–80 m, 9–23 vii 1987, V.O. Becker & S.E. Miller (3♂, 1♀); Col. Becker 66649, Brit. Virgin Isl., Guana I, 0–80 m, 9–23 vii 1987, V.O. Becker & S.E. Miller (2♂, 5♀); same as previous, Genitalia 1296 [Abdomen remains on specimen (!)] (1♂); Brit. Virgin Isl., Guana Island, 0–80m, 13–26 July 1986, S.E. Miller & M.G. Pogue, North Bay, *Coccoloba* forest, U.V. light trap. sea level, 15–25 July 1986, [note label dates contradict], #25 (1♂); Brit. Virgin Isl., Guana I, 0–80 m, 24–31.x. 1990, S.E. Miller & T.M. Kuklenski, Collectors Bishop Museum, n. gen. + sp, Schacontiinae det. E.G. Munroe, 1991, Schacontia n. sp. det. M.A. Solis (1♂); Brit. Virgin Isl., Guana I, 0–80 m, 10–25.vii.1988, S.E. Miller & C. O’Connell, Colls. Bishop Museum, 89: 13 Apr #1 EGM (1♂); Brit. Virgin Isl., Guana Island, 1–14 July 1984, S.E. & P.M. Miller, 39, Genitalia Slide by JAL ♀ USNM 108,878 (1♀); Anomalous BVI specimens with tibial hair pencils but no abdominal coremata (3♂): Col. Becker 70821, Brit. Virgin Isl., Guana X. 1989 V.O. Becker, 23, Genitalia slide ♂ by JAL (1♂); Col. Becker 66650**,** Brit. Virgin Isl., Guana I., 0–80 m 9–23.vii.1987 V.O. Becker & S.E. Miller, 13, Genitalia slide ♂ by JAL (1♂); Brit. Virgin Isl., Guana Island, 0–80 m, 5–23 July 1985, S.E. & P.M. Miller, Clubhouse, 60 m, U.V. light trap, 9–15 July 1985, 38, VOB ♂ USNM 108,877 (1♂). **Mexico** (4♀). Chichen Itza. Yucatan, Mexico. EC Welling Coll. 5.III.1956 (1♀) [CNC]; Chichen Itza, Yucatan, Mexico, EC Welling Coll., 2.III.956 (1♀) [CNC]; Mexico, Mazatlan, July 10, 1969, El 50’, James H. Baker collection 1978 (1♀); Taboga Isl Pan, Febr 12 August, Busck (1♀). **United States** (1♀): Florida. “Sanibel Island, Lee Co., FLA.”, “30-VI-1984 Leg. W.L. Adair”, MGCL slide no. 687 [MGCL].

##### Diagnosis.

*Habitus* (Fig. 6). Unlike many *Schacontia*, there is little to no contrast between the medial area and the rest of the forewing; although this holds for both *Schacontia rasa* and *Schacontia clotho* as well, those two are readily distinguished on other grounds. Male *Schacontia themis* exhibit the full range of secondary sexual features known from the genus (flattened hind tibial spur, elongate hind tibial scales with embedded dark patch, epipleural setae, and concave metatarsal structure) as well as long abdominal coremata (Fig. 19; Table 1); *Schacontia rasa* (below), the putative sister species of *Schacontia themis*, has none of these (but see remarks). Two other *Schacontia* species (*Schacontia clotho* and *Schacontia lachesis*) do share these features in part, but not the genitalic configuration that characterizes *Schacontia themis* and *Schacontia rasa* (see below). *Male and female genitalia* (Figs 49–51). As with the remaining *Schacontia*, males of this species are most readily diagnosed by a combination of genitalic and external secondary sexual characteristics. The male genitalia best distinguish this species and its putative sister, *Schacontia rasa* (below), from other *Schacontia*: The uncus has a characteristic, expansive trefoil-shaped tip and lateral edges that appear swollen or re-enforced (as *Schacontia lachesis* and *Schacontia atropos* do), and a raised, pronounced medial ridge (as they do not). The intrasaccular flange is robust and forms a trigger-shaped process at the latero-ventral edge of the valva.

**Table 1.** Species diagnoses for the *Schacontia nyx* complex based on male genitalia and secondary sexual characters. Format inspired by Ferguson (1992: 259).<br/>

	Thorax					Abdomen	Genitalia			
	Flattened hind tibial spur	Elongatehind tibial scales	Dark patch embedded within tibial scales	Epipleural setal tufts	Concavemeta-tarsal structure	Coremata	Saccular bend or ulna	Ventro-medial setal comb	Distribution of ventro-marginal setae	Hook-shaped decumbent costal setal cluster
themis	+	0/+	0/+	+	+	Short	NA	0	0	0
rasa	0	0	NA	0	0	NA	NA	0	0	0
nyx	+	+	0	0	+	Short	Round	+	Localized	0
clotho	+	+	+	+	+	Long	Round	+	Localized	+
lachesis	+	+	0/+	+	+	Short	Angled	0	Even	+
atropos	0	0	NA	0	0	NA	Angled	0	Even	+

##### Description.

Male. (Fig. 6). Forewing length: 5.3–10.0 mm. **Head** - Ocelli present; proboscis with pale basal scales in males, light brown basal scales in females. Vertex and frons yellowish white in males, intermixed with brownish-gray scales in females; frons of normal (convex) contour; maxillary palpi uniformly grayish brown; labial palpi grayish brown in males, fading to gray apically in females; labial and maxillary palpi porrect, extending well beyond clypeus; antennal scape and pedicel yellowish white, flagellomeres grayish brown. **Thorax** - Thoracic collar, tegula and mesoscutum yellowish white in males, brownish gray in females. Males with flattened hind tibial apical spur, hind tibial tuft of long black scales intermixed with pale yellowish-white scales, epipleural setae, and shallow concave metatarsal modification. Females with two pair of hind tibial spurs (medial pair present). Forewingground color straw, with few contrasting markings other than jagged chocolate-brown antemedial and postmedial lines, postmedial line outwardly bulging only slightly, towards costa. Medial area unicolorous with basal area and postmedial areas. Fringe darkened apically. *Hindwing*. Almost uniformly pale, shaded brown at subterminal area. **Abdomen** - Dorsal surface straw brown. Scales arranged in two terminal black dorsal spots in males. Lobe-like extensions resembling rudimentary coremata on 4^th^ abdominal sternum. *Tympanal organs*. (Fig. 26). As for *ysticalis-themis* group, *vide supra*. **Male genitalia** (Figs 49, 50) - Tegumen divided into two obliquely opposed oval sections meeting caudally near base of uncus and diverging widely anteriorly towards valvae. Teguminal sulcus short, such that anterior margin of tegumen appears deeply invaginate, two oblong teguminal lobes joined obliquely. Uncus with prominent medial ridge; uncal tip hastate, trefoil-like; lateral edges of uncus swollen, appearing re-enforced and lending a shovel- or scoop-like appearance to uncus. Gnathal plate narrowed to a transverse band with arms at each corner and a small, nub-like process arising centrally; dorsal arms wrap around anal tube while a ventral pair extend to termini of vinculum. Vinculum variously U-shaped or horseshoe shaped with pronounced pockets or eyelets at each terminus. Juxta robust, V-shaped; valvae complex, robust costa arising near vincular terminus, extending almost length of valva, and tapering to a point. A serrate, trigger-like ventral spine arises from ventral margin of sacculus; robust, spine-like setae at base of valva; saccular margin angled close to vinculum, not at saccular mid-point; ventro-marginal setae concentrated at venter or saccular ulna; valva with secondary fleshy subcostal setose lobe, setal plume recurved/decumbent. Elongate saccular process at outer saccular margin; saccular margin with several stout setae. Inner surface of valvae dorsal to saccular area with a small, circular setal cluster. Phallus moderately sclerotized; vesica with two large cornuti. **Female genitalia** (Fig. 51) - Papillae anales separate, not especially swollen; anterior and posterior apophyses threadlike, approximately equivalent in length; antrum/ductus bursae conspicuously sclerotized with a partial ventral collar or sleeve ventrally anterior to colliculum; a sharp constriction between ductus bursae and colliculum; colliculum a short sclerotized ring immediately posterior to corpus bursae with a narrow differentially sclerotized band around its center; corpus bursae membranous, more or less round, without signa or conspicuous appendices except single posterio-dorsal lobe; ductus seminalis appears to originate from antrum.

##### Immature stages.

Unknown.

##### Variation.

This species varies most obviously in size (5.3 mm–10.0 mm in male forewing length), and based on the examination of several anomalous specimens from the British Virgin Islands, the presence of male secondary sexual characteristics (tibial hair pencils and abdominal coremata) do not perfectly covary: Hair-penciled males with and without coremata are noted from Guana Island, examples annotated and/or segregated in “Material examined” section above; see also Discussion.

##### Etymology.

The specific epithet refers to the Greek Titaness and embodiment of divine order and is treated as a noun in apposition.

##### Biology.

Possibly associated with Capparaceae. (http://janzen.sas.upenn.edu/caterpillars/database.lasso). Phenology, based on examined material: January–April, August in Costa Rica; March–April in Panama; April–May in Jamaica & Cayman Islands; July in Dominican Republic; June–July in Cuba; June, August in Puerto Rico; April–May in Venezuela; July–August in Brazil.

##### Distribution.

Brazil, Cayman Islands, Costa Rica, Cuba, Dominican Republic, Florida (USA), Jamaica, Mexico, Panama, Puerto Rico, Venezuela. The Sanibel Island, Florida record represents the only known United States occurrence of any *Schacontia*.

##### Remarks.

*Schacontia themis* is among the more widespread and collected species of *Schacontia*. It seems likely that more cryptic species (like *Schacontia rasa*, below) exist.

#### Schacontia rasa

Solis & Goldstein sp. n.

urn:lsid:zoobank.org:act:FDC54534-0A71-424E-9CFC-C48604E452F5

http://species-id.net/wiki/Schacontia_rasa

Figs 7 , 27 , 52–54

##### Material examined.

(22♂, 32♀).

##### Type material.

(3♂, 13♀), USNM. Holotype ♂ (Fig. 7). **Mexico**: Col. Becker 110514; Mexico: Tam San Fernando, 50 m, 28. vi. 1997, V. O. Becker Col.; USNM genitalia slide “JAL 18” Paratypes (2♂, 13♀, 4 sex undet.). Same data as holotype (2♂, 4 sex undet., 13♀ incl.1 with green USNM genitalia slide label “JAL 19.”

##### Other material examined.

**Cuba** (3♂, 2♀):Col. Becker 72733, Cuba Gtnmo. Imias, 10 m, 17. vii. 1990, V.O. Becker, USNM ENT 00808543, DNA 2012 (1♂); Col. Becker 72409, Cuba: Holquin Mayari, 400 m, 12.vii.1990, V.O. Becker, USNM ENT 00808544, DNA 2012 (1♀); Santiago, Cuba, Collection Wm Schaus, Genitalia slide by DA ♂ USNM 108,096 (1♂); Col. Becker 73068, Cuba: Stgo. Siboney, 20 m, 23.vii.1990, V.O. Becker, genitalia slide by JAL, 16 (1♂, 1♀). **Dominican Republic** (15♂, 13♀) [CMNH]: Dominican Republic: Pedernales, 14.5 km N Cabo Rojo, 18-03N, 71-39W, 165 m, 19 July 1990, J. Rawlins, C.W. Young, S.A. Thompson (1♂, 7♀); Dominican Republic: Pedernales, 9.5 km N Cabo Rojo, 18-02N, 71-39W, 35 m, 19 July 1990, J. Rawlins, C.W. Young, S.A. Thompson (5♂, 3♀); Dominican Republic: Pedernales, 14.5 km N Cabo Rojo, 10 m, 17-55N, 71-39W 26–27 September 1991, C. Young, B. Thompson, R. Davidson, J. Rawlins Coastal desert (2♂, 1♀); Dominican Republic: Pedernales, 14.5 km N Cabo Rojo, 18-03N, 71-39W, 165 m, 26–27 September 1991, C. Young, S. Thompson, R. Davidson, J. Rawlins, Arid thornscrub (1♂); Dominican Republic: Pedernales, 1 km W Cabo Rojo, 17-55N, 71-39W, 10 m, 30 July 1990, C.W. Young, J.E. Rawlins, S. Thompson (4♂, 1♀); Dominican Republic: Pedernales, Cabo Rojo, Sea level, 17-55N 71-39W, 21 Oct 1991, J. Rawlins, R. Davidson, C. Young, S. Thompson, Edge of salt marsh (1♂); Dominican Republic: La Altagracia, Parque del Este, Caseta Guaraguao, 4.4 km SE Bayahibe, 18-19-59N, 68-48-42W, 3 m, 26–27 May 2004, C. Young, J. Rawlins, J. Fetzner, C. Nunez, semi-humid forest near sea, limestone, UV light. Sample 51114 (1♀); Dominican Republic: La Altagracia, 2 km N Bayahibe, 10-23N, 68-51W, 10 m, 3 July 1992, C. Young, R. Davidson, S. Thompson, J. Rawlins, Dry seasonal forest on limestone, USNM ENT 00808542, DNA 2012 [GenBank Accession #KC789514] (1♂). **British Virgin Islands** (1♂, 2♀):Virgin Gorda BVI Prickley Pear Id, Vixen Pt, 14.IV.56, J.F.G. Clarke, 33, Genitalia Slide ♂ by JALUSNM 108,872 (1♂); Col. Becker 66649, British Virgin Is., Guana I., 0–80 m. 9–23 vii 1987, V.O. Becker & S.E. Miller, #20, Genitalia Slide ♀ by JAL (1♀); British Virgin Is.,Virgin Gorda Island, V. Gorda Peak, Ca. 400 m, 17–19 July 1986, S.E. Miller & M.G. Pogue, Black light trap in secondary moist forest, 37, Genitalia slide by JAL ♀ USNM 108,876 (1♀).

##### Diagnosis

(Figs 7, 52–54). Very similar to *Schacontia themis* (above), particularly with respect to the male genitalia, but lacking the secondary sexual characters enumerated above and the forewing ground color usually mousegray instead of straw colored. Female hind tibia with a single pair of spurs (medial pair absent - diagnostic within the *ysticalis-themis* group).

##### Description.

Male (Fig. 7). Forewing length: 7.0 mm–8.0 mm. **Head** - Ocelli present; proboscis normal; frons unmodified; labial palpi porrect, extending beyond clypeus. **Thorax** - *Forewing*. Medial area gray, unicolorous with basal area and postmedial areas; antemedial and postmedial lines jagged, darker gray. *Hindwing*. Postmedial line faint if present; outwardly tinged with bronze. **Abdomen** - Terga unicolorous with wings and thorax; scales arranged in two terminal black dorsal spots in males. *Tympanal organs*. (Fig. 27). As for *ysticalis-themis* group, *vide supra*. **Male genitalia** (Figs 52, 53) - Teguminal sulcus short, anterior margin of tegumen appears deeply invaginate, two oblong teguminal lobes joined obliquely; uncus trefoil shaped, outermost tip hastate; lateral edges of uncus swollen, appearing re-enforced; uncus with raised, pronounced medial ridge; juxta robust, V-shaped; valvae complex, intrasaccular flange at latero-ventral edge and sclerotized to form a trigger-shaped process; robust, spine-like setae at base of valva; saccular margin angled close to vinculum, not at saccular mid-point; ventro-marginal setae concentrated at saccular ulna; valva with secondary outer, oblong lobe or process below costa; fleshy setose lobe and recurved/decumbent setal plume associated with terminus of costa. Phallus moderately sclerotized; vesica with two large cornuti. **Female genitalia** (Fig. 54) - Very similar to *Schacontia themis* but posterior lobe of corpus bursae more pronounced, superficially rugose; both colliculum and sclerotized channel or sleeve of ductus more elongate than in *Schacontia themis*.

##### Immature stages.

Unknown.

##### Etymology.

The specific epithet refers to the absence of male hind tibial and metatarsal structures and epipleural setal tufts (presumably secondary sexual characters) present in other *Schacontia* species (Table 1).

##### Biology.

Unknown. Adults active June (Mexico), July (Cuba), and July, September (Dominican Republic).

##### Distribution.

Mexico, Cuba, Dominican Republic (essentially, vic. Gulf of Mexico).

##### Remarks.

*Schacontia rasa* is evidently the sister species of *Schacontia themis*. Were it not for the characters associated with the forewing ground color, female hind tibia, and male genitalia and given the homoplastic nature of certain male secondary sexual characters comparable to the system described by Ohno (2000) [see discussion], *Schacontia rasa* would be a more obvious candidate for conspecificity with *Schacontia themis*. We have treated anomalous specimens with “chimeric” distributions of male secondary sexual chacters under *Schacontia themis* (above), and considered only those lacking both tibial hair pencils and abdominal coremata (in addition to genitalic features) to be unambiguously *Schacontia rasa*, recognizing the need for future molecular work to evaluate the degree to which these character systems are functionally and genetically linked. DNA barcode data (meeting the Barcode Data Standard of Genbank, noted in Benson et al. 2012) are limited to three Dominican Republic specimens and do decisively unite two specimens of *Schacontia themis* to the exclusion of *Schacontia rasa*. Not enough specimens are sampled to test the variable sites as diagnostic characters and enable their use in the species’ diagnoses (Goldstein and DeSalle 2011), but the data corroborate (albeit by a distance measure of >7%) the reliability of the morphological characters as consistent with two distinct species.

It was suggested by V.O. Becker (pers. comm., following the submission of this work) that the name *Dichogama fernaldi* Möschler, 1890, the type material of which has apparently been lost from MNHU, might refer to this species (see Becker & Miller, in prep., for discussion) and that it should be placed in the now monotypic genus *Dichochroma*, whose description is, in turn, based on a single female (and only known specimen) of the type species, *Dichochroma muralis* Forbes, 1944. This attribution of the specimens we consider to fall within *Schacontia themis* (or *Schacontia rasa*, below) to *Dichochroma fernaldi* is, however, not well corroborated by any character identified in the original description by Möschler, but only by process of elimination and the report of its being reared on *Capparis* by Wolcott (1950-1951: 658, cited in Becker and Miller, in prep.). While eliminating a *nomen dubium* is desirable and the process of elimination by which such an attribution might be reached intriguing, the recognition of two similar co-occurring species (*Schacontia themis* and *Schacontia rasa*) described here, corroborated at least indirectly by DNA barcode data, precludes any specific attribution. We therefore retain the description of *Schacontia themis* and *Schacontia rasa* as such. Further, notwithstanding the superficial similarity of certain female *Schacontia* genitalia to those of the only known specimen *Dichochroma muralis*, both phylogenetic placement described in this work and priority of *Schacontia* would dictate that *Dichochroma* be sunk were it determined that these species were congeneric, even if male *Dichochroma muralis* were discovered and or more compelling character data were brought to bear.

**The Schacontia nyx complex:** Some of these species are not readily diagnosed by a single character; each, rather, is characterized by either an absence of characters (as in *Schacontia atropos*) or by combinations of characters, all of them male, both genitalic and external, the latter presumably secondary sexual features.

#### Schacontia nyx

Solis & Goldstein sp. n.

urn:lsid:zoobank.org:act:B7C1FDB3-9A23-44BB-9CD4-8E1D8B8DBE78

http://species-id.net/wiki/Schacontia_nyx

Figs 8 , 28 , 33 , 55–57

##### Material examined.

**Type material.** Holotype (♂, USNM): **Venezuela**: Guarico, Hato Masaguaral, 45 km S Calabozo 8.57N, 67.58W, Galry For #10, 75 m, 23–24 April 1988 uv lt. M. Epstein & R. Blahnik. Paratypes (4♂, 2♀), USNM: Venezuela: Same data as holotype (1♂, 1♀); Guarico, Hato Masaguaral, 45 km S Calabozo 8.57N, 67.58W, Galry Forest #20, 75 m, 13–16 May 1988 uv lt. M. Epstein & R. Blahnik (2♂, 1♀); Lara, 4 km NW of La Pastora 2–3 III 1978 riparian forest blacklight, J.B. Heppner, Genitalia slide by DA ♂ USNM 108,101 (1♂)

##### Diagnosis.

*Habitus* (Fig. 8). Although more readily diagnosed by the male genitalia, *nyx* can be differentiated on the basis of wing pattern. *Nyx* shares with other members of the genus (and in particular of the complex of sibling species to which it belongs) the configuration of the medial area, with its outward subcostal bulge, but its more mottled appearance and less uniformly contrasting ground coloration between the medial and both the antemedial and postmedial areas. Male *Schacontia nyx* does not bear the epipleural setae shared by most other members of the complex; nor do they exhibit a dark patch embedded within the elongate hind tibial scales (Table 1).*Genitalia* (Figs 55, 56). Male specimens of *Schacontia nyx* are most readily diagnosed by the obovate uncus, which is without pronounced medial ridges or lateral swellings. The subcostal processes are more conspicuous and elongate than in *Schacontia themis* or *Schacontia rasa*, but less narrow than in other species in the complex (*Schacontia clotho*, *Schacontia lachesis*, *Schacontia atropos*, or *Schacontia androgyne*).

##### Description.

Male (Fig. 8). Forewing length: 6.0–7.0 mm (n=3) (Female 7.3–7.7 mm; n=2). **Head** - Ocelli present; proboscis normal; frons of normal contour; labial and maxillary palpi drooping, extending beyond clypeus; vertex gray. **Thorax** - Tegulae uniformly gray brown; flattened hind tibial spur, specialized hind tibial scales, and shallow concave metatarsal modification all present; female with two pair of hind tibial spurs (medial pair present). *Forewing*. Antemedial and postmedial lines darkened at medial area, white towards basal and postmedial areas; white scales suffused both in basal area near anal margin and medial area, especially surrounding orbicular spot; patchy white scales in postmedial area; subterminal line unbroken; FW fringe dark brown, scales paler basad. *Hindwing*. White, shaded grayish brown towards margin; postmedial line absent; subterminal line unbroken; HW fringe gray, scales paler basad. **Abdomen** - Scales arranged in two terminal black dorsal spots in males. Short coremata on 4^th^ abdominal segment (Fig. 33). *Tympanal organs* (Fig. 28). As for *ysticalis-themis* group, *vide supra*. **Male genitalia** (Figs 55, 56) - Teguminal sulcus short, anterior margin of tegumen appears deeply invaginate, two oblong teguminal lobes joined obliquely; uncus trefoil-shaped tip reduced to a small, more or less rhomboid nipple, edges simple, undifferentiated; juxta U-shaped, tapered ventrally; valva complex, intrasaccular flange at latero-ventral edge and sclerotized to form a trigger-shaped process; robust, spine-like setae at base of valva; saccular margin rounded at saccular mid-point; prominent setal comb at ventro-medial margin of valva; ventro-marginal setae concentrated at saccular ulna; valva with pronounced, elongate secondary outer lobe or process below costa; recurved/decumbent setal plume associated with terminus of costa. Phallus moderately sclerotized; vesica with two large cornuti. **Female genitalia** (Fig. 57) - Papillae anales separate, not especially swollen; antrum/ductus bursae present, diffusely sclerotized ventrally anterior to colliculum; colliculum present, short, sclerotized, immediately posterior to corpus bursae, with narrow differentially sclerotized band around center; ductus seminalis attached posterior of corpus bursae; corpus bursae round; small accessory bursa present; ductus seminalis originates at posterior end of corpus.

##### Immature stages.

Unknown

##### Etymology.

*Nyx*, the primordial goddess of the night who according to myth stood at the beginning of creation, refers to the first of five closely related species in this complex. The specific epithet is treated as a noun in apposition.

##### Biology.

Unknown. Adults March–May.

##### Distribution.

Northern Venezuela.

#### Schacontia clotho

Solis & Goldstein sp. n.

urn:lsid:zoobank.org:act:55C68C29-D157-4838-8F52-F277B9E1CA66

http://species-id.net/wiki/Schacontia_clotho

Figs 9 , 29 , 34 , 58–60

##### Material examined.

**Type material.** Holotype (♂, USNM) (Fig. 9): **Ecuador**: Loja Catamayo, 1300 m, 20. xii 1992, V.O. Becker Col., Col. Becker 102660, Genitalia 1287. Paratypes (1♂, 1♀), USNM. Ecuador: Same data as holotype (1♂, 1♀), the latter accompanied by “Genitalia Slide ♀ by JAL.”

##### Diagnosis.

*Habitus* (Fig. 9). This species superficially resembles *Schacontia rasa* in coloration and maculation; it is smaller and the male bears all of the secondary sexual characters, including coremata, known to occur within *Schacontia* (Table 1).*Genitalia*(Figs 58–60). The male genitalia of *Schacontia clotho* place it unambiguously within the *Schacontia nyx* complex as opposed to with *Schacontia themis* or *Schacontia rasa*. Moreover the subcostal lobe of the valva is elongate.

##### Description.

Male. (Fig. 9). Forewing length: 6.9–7.0 mm (n=3) (Female 6.8 mm). **Head** - Ocelli present; proboscis normal; frons of normal contour; labial palpi porrect, extending beyond clypeus. **Thorax** - Prothoracic collar and tegulae an admixture of brown and mouse-gray scales. Flattened hind tibial spur, specialized hind tibial scales, epipleural setae present, and dark patch amidst male hind tibial scales all present. Female with two pairs of hind tibial spurs (medial pair present); shallow concave metatarsal modification present. *Forewing*. More lanceolate than in other *Schacontia* species. More or less uniform mouse gray, with very light dusting of very pale gray in medial and postmedial areas; medial area more darkly shaded than basal area and postmedial areas; FW fringe brown; subterminal line unbroken. *Hindwing*. Nearly translucent; postmedial line absent; fringe pale yellowish; subterminal line unbroken. **Abdomen -** Scales arranged in two terminal black dorsal spots in males; elongate coremata on 4^th^ abdominal segment (Fig. 34). *Tympanal organs* (Fig. 29). As for *ysticalis-themis* group, *vide supra*. **Male genitalia** (Figs 58, 59) - Uncus trefoil-shaped tip reduced to a small, more or less rhomboid nipple; lateral edges of uncus simple, undifferentiated; juxta broadly V-shaped, comparable in shape to an avian furcula, arms not robust; valvae complex, intrasaccular flange at latero-ventral edge and sclerotized to form a trigger-shaped process; robust, spine-like setae at base of valva; saccular margin rounded at mid-point; prominent setal comb at ventro-medial margin of valva; ventro-marginal setae concentrated at saccular ulna; costal bar diverges from subcostal lobe towards base of costa (isolation of costa >75% along length, character 49); valva with pronounced, elongate secondary outer lobe or process below costa; recurved/decumbent setal plume associated with terminus of costa; sharply hooked setal cluster prominent. Phallus moderately sclerotized; vesica with two large cornuti. **Female genitalia** (Fig. 60) - Papillae anales separate, not swollen; antrum/ductus bursae elongate (not chalice-like), faintly sclerotized posterior to colliculum, separated from colliculum by a sharp constriction; colliculum a short ring (not an elongate collar), with faintly sclerotized band, immediately posterior to corpus bursae; corpus more or less globular, surface complex; ductus seminalis originates at posterior end of corpus bursae.

##### Immature stages.

Unknown.

##### Etymology.

The specific epithet refers to the youngest of the three fates in Greek mythology, responsible for spinning the thread of human life, and is treated as a noun in apposition.

##### Biology.

Unknown. Adults December.

##### Distribution.

Southern Ecuador.

#### Schacontia lachesis

Solis & Goldstein sp. n.

urn:lsid:zoobank.org:act:886A4F51-EA8A-4D43-9C58-FA7A1743BA1A

http://species-id.net/wiki/Schacontia_lachesis

Figs 10, 11 , 30, 35 , 61–63

##### Material examined.

**Type material.** Holotype (♂, USNM) (Fig. 10): **Brazil:** Col. Becker 55439, Brasil: RJ Arrai al do Cabo, 50 m, 29.i.1985, V.O. Becker col. Paratypes 14 males, 11 females, USNM: **Brazil:** Same data as holotype (1♂, 1♀);Col. Becker 91635, Brasil: CE Pacatuba, 250 m, 6. iv. 1994, V.O. Becker Col. (1♂);Col. Becker 111257, Brasil: MT 60 km, S Poconé, 1–7.xii.1997, V.O. Becker Col. (1♀);Col. Becker 88540, Brasil: RO Cacaulãndia, 140 m, 15–18.x.1993, V. O. Becker Col. (1♀);Col. Becker 93888, Brasil: MT Chapada dos Guimarães, 800 m, 20.xi.1994, V. O. Becker Col. (1♀);Col. Becker 105713, Brasil: BA Jequié, 600–750 m, 11–22. xi.1995, V.O. Becker Col. (4♂, 1 ♀ and a disassociated male genitalic slide); Col. Becker 54553 Brasil: RJ Maricá, 5 m, 12–15.i.1995, V.O. Becker Col. (3♂, incl. 1 male w/white tag “Genitália 1259”, 1♀). **Bolivia**: Santa Cruz, Puerto Suarez, 150 m, Nov 1908, J. Steinbach, CMNH Acc. 3758 (3♂, 1 abd. detached, prob. ♀); Santa Cruz, Provincia del Sara, 350 m, October 1911, Jose Steinbach, CMNH Acc. 5038 (2♀); Santa Cruz, Provincia del Sara, 350 m, Dec 1912, Jose Steinbach, CMNH Acc. 5038 (1♀); Santa Cruz, Puerto Suarez, 150 m, Dec 1908, J. Steinbach, CMNH Acc. 3758 (2♂, 1♀, dissection).

##### Diagnosis.

*Habitus* (Figs 10–11). A polymorphic species, some specimens resembling *Schacontia rasa* and *Schacontia clotho* in showing little to no ground color contrast between the variably mouse-gray or straw-colored medial area and the rest of the forewing; other specimens display a sharper contrast, with the medial area primarily gray and the antemedial and postmedial areas straw colored, very similar to some specimens of *Schacontia atropos* (below). Like most members of the genus (other than *Schacontia rasa*; Table 1), *Schacontia lachesis* males bear specialized hind tibial scales, but like *Schacontia nyx* some specimens lack the dark patch embedded within them. *Genitalia* (Figs 61–63).Like those of *Schacontia themis*, *Schacontia rasa*, and *Schacontia atropos*, male genitalia of *Schacontia lachesis* have the uncus with raised or swollen edges, but do not share the other synapomorphies of the *Schacontia themis*-*rasa* pair and strongly resemble other members of the *Schacontia nyx* complex in bearing more elongate subcostal processes and ornate costae. Males of *Schacontia lachesis* are distinguished from those of *Schacontia atropos* (below), which they most closely resemble, by a combination of short coremata (Fig. 35), angled as opposed to rounded saccular bend (ulna), absence of a ventro-medial setal comb, and a more even distribution of ventro-marginal setae (Table 1).

##### Description.

(Figs 10, 11). Male. Forewing length: 5.0–7.5 mm. **Head** - Ocelli present; proboscis normal; frons of normal convex contour; labial palpi porrect, extending beyond clypeus. **Thorax** - Flattened hind tibial spur, specialized hind tibial scales, and epipleural setae present; female with two pair of hind tibial spurs (medial pair present); shallow concave metatarsal modification. *Forewing*. Prothoracic scaling tan gray, straw or yellowish. Forewing coloration equally variable, medial area polymorphic, exhibiting a range of contrast not known from other *Schacontia*, ranging from light to dark brown in both sexes, dusted with white; basal and postmedial areas straw colored; antemedial and postmedial lines jagged; orbicular spot faint but apparent; fringe scales darker at base; subterminal line unbroken. *Hindwing*. Postmedial line absent; fringe scales darker at base; subterminal line unbroken. **Abdomen** - Coremata on 4^th^ abdominal segment (Fig. 35); scales arranged in two terminal black dorsal spots in males. *Tympanal organs* (Figs 30, 31). As for *ysticalis-themis* group, *vide supra*. **Male genitalia** (Figs 61, 62) - Teguminal sulcus short, such that anterior margin of tegumen appears deeply invaginate, two oblong teguminal lobes joined obliquely; lateral edges of uncus swollen, appearing reinforced; uncus trefoil-shaped tip reduced to a small, more or less rhomboid nipple; juxta U-shaped, tapered ventrally; valvae complex, intrasaccular flange transposed towards latero-ventral edge and sclerotized to form a trigger-shaped process; robust, spine-like setae at base of valva; saccular margin sharply angled at saccular mid-point; ventro-marginal setae distributed along length of outer margin of sacculus; valva with pronounced, elongate secondary outer lobe or process below costa; fleshy setose lobe and recurved/decumbent setal plume associated with terminus of costa; sharply hooked setal cluster prominent. Phallus moderately sclerotized; vesica with two large cornuti. **Female genitalia** (Fig. 63) - Papillae anales separate, unswollen; colliculum present, short, sclerotized, immediately posterior to corpus bursae, with narrow sclerotized band around center, sometimes separated from bursa by a sharp constriction; ductus bursae present, conspicuously sclerotized ventrally, entering corpus bursae dorsally; appendix bursae ventral, superficially complex; ductus seminalis attached at posterior end of ventral corpular out-pocketing.

##### Immature stages.

Unknown.

##### Etymology.

In Greek mythology, *Lachesis* is the middle sister of the three fates, the personification of destiny responsible for measuring the duration of human life. The specific epithet is treated as a noun in apposition.

##### Biology.

Unknown. Adults in Brazil active January, April, November, December; adults in Bolivia active October–December.

##### Distribution.

Central Brazil (Rondonia east to Bahia, Ceara and Rio de Janeiro), Bolivia (Santa Cruz).

#### Schacontia atropos

Solis & Goldstein sp. n.

urn:lsid:zoobank.org:act:D7D8AE98-0E23-4C74-8813-79F0B16CBF62

http://species-id.net/wiki/Schacontia_atropos

Figs 12 , 32 , 64, 65

##### Material examined.

**Type material.** Holotype (♂, USNM) (Fig. 12): **Venezuela**: San Estaban Carabobo, Venez., Dec. 1–20 1939, Pablo J. Anduse. Paratypes (2♂), USNM. **Venezuela**: Same data as holotype (1♂); Lara, 4 km NW of La Pastora, 2–3 III 1978 riparian forest blacklight, J.B. Heppner (1♂).

##### Diagnosis.

*Habitus* (Fig. 12). Overlaps in appearance with *Schacontia lachesis*, but males lack all secondary sexual features; hindwing uniformly pale throughout. *Genitalia* (Figs 64, 65). Male genitalic features place it squarely in the *nyx* complex, from whose other member species it may be distinguished by the combination of the angled ventral edge of the saccus (ulna) and unmodified edges of the uncus.

##### Description.

(Fig. 12). Male. Forewing length: 5.4–5.5 mm (n= 2). **Head** - Ocelli present; proboscis normal; frons of normal contour; labial palpi porrect, extending beyond clypeus. **Thorax** - Prothoracic scaling light straw yellow. Female with two pair of hind tibial spurs (medial pair present). *Forewing*. Ground color staw yellow; medial area brown gray, heavily suffused with white scales, orbicular spot present. *Hindwing*. Pale overall, nearly translucent, not more darkly tinged subterminally as in other members of *Schacontia nyx* complex; postmedial line faint if present. **Abdomen** - Scales arranged in two terminal black dorsal spots in males. *Tympanal organs* (Fig. 32). As for *ysticalis-themis* group, *vide supra*. **Male genitalia** (Figs 64, 65) - Teguminal sulcus short, such that anterior margin of tegumen appears deeply invaginate, the two oblong teguminal lobes joined obliquely; uncus trefoil-shaped tip reduced to a small, more or less rhomboid nipple; juxta U-shaped, tapered ventrally; valvae complex, intrasaccular flange transposed towards latero-ventral edge and sclerotized to form a trigger-shaped process; robust, spine-like setae at base of valva; saccular margin sharply angled at saccular mid-point; ventro-marginal setae distributed along length of outer margin of sacculus; valva with pronounced, elongate secondary outer lobe or process below costa; fleshy setose lobe and recurved/decumbent setal plume associated with terminus of costa; sharply hooked setal cluster prominent. Phallus moderately sclerotized; vesica with two large cornuti. **Female genitalia** - Unknown.

##### Immature stages.

Unknown.

##### Etymology.

The specific epithet refers to the third of the three fates. Treated as a noun in apposition.

##### Biology.

Unknown.

##### Distribution.

Northern Venezuela.

##### Remarks.

Given the phenomenon described by Ohno (2000) it is well within the realm of possibility that this species represents a synonym of *Schacontia lachesis*.

## Discussion

The moths treated in this paper compise a range of geographically widespread and potentially localized cryptic species united by a range of synapomorphies. It is not possible to infer an unambiguous center of origin for *Schacontia*; members of both the typical *medalba* group and the *ysticalis-themis* group are distributed from Mexico to South America. While we note at least three trans-isthmian species (*Schacontia chanesalis*, *Schacontia ysticalis*, and *Schacontia themis*), several species - including the entire *Schacontia nyx* complex - are restricted to South America, while *Schacontia rasa* is known only from Mexico and the Caribbean. Among the more intriguing features of *Schacontia* that warrant further study is their gall-forming habits in association with capparaceous plants, and the enormous variation in size, which may be a function of indeterminate instar number.

There likely exist undiscovered *Schacontia* species, cryptic and otherwise. *Schacontia chanesalis* in particular may represent a complex of Mayrian species that are difficult to diagnose without more extensive molecular data. However, available data are such that obvious breaks in the continuum of variation are not obvious, and rather than allowing the weakly articulated epithet *Schacontia replica* to persist as valid we have elected to synonymize it, along with *Schacontia pfeifferi*. Although its presumptive range differs geographically from that of S. chanesalis, we saw little point in retaining *Schacontia pfeifferi* given a lack of apparent distinguishing characters. Although its presumptive range differs geographically from that of *Schacontia chanesalis*, we saw little point in retaining *Schacontia pfeifferi* given a lack of apparent distinguishing characters. We took a less conservative approach within the *Schacontia nyx* complex, nominating species on the basis of characters we suspect may be more labile than our limited collection of specimens suggests. We attribute the lack of phylogenetic decisiveness, particularly in the *nyx* complex, to homoplasy among characters associated with the male secondary sexual features.

By far the richest source of phylogenetic signal in our matrix are the male genitalia, accounting for almost half the characters included in our analysis. This is not unusual for species-level studies of Lepidoptera (or insects generally), and there may be a growing consensus that despite their likely being subjected to sexual selection and thus potentially evolving quite rapidly (Fisherian runaway), male genitalic characters nonetheless contain valuable information at multiple hierarchic levels (Simonsen and Roe 2009; Song and Bucheli 2010), a situation analogous to that of third positions or transitions in molecular phylogenetics (e.g. Källersjö et al. 1998). A noteworthy exception is the work of Solis and Maes (2002), who concluded that male genitalic characters were not especially useful at the subfamily level within the Crambidae.

In contrast, it is of particular relevance to the taxonomy of *Schacontia* that relatively little complex variation is observed in the female genitalia, particularly given that four of the five species described prior to this work - two of which are synomized herein - have female holotypes.

Solis et al. (2009) discussed the roles of secondary sexual structures, including scent-producing structures and their associated modified scales (reviewed by Hallberg and Poppy 2003), in lepidopteran diversification broadly and in Pyraloidea specifically. They highlighted the historical differences of opinion between taxonomists who discounted the importance of such structures and those who viewed them as invaluable in lepidopteran classification (e.g., Janse 1931) and, following Solis (1993) and Simonsen and Roe (2009), in phylogenetic reconstruction in spite of empirical demonstrations of homoplasy. Based on the present work, male secondary sexual characters were among the more homoplastic we analyzed, at least insofar as their removal contributes resolution to the phylogenetic hypothesis generated. The intraspecific lability of characters such as hind tibial hair pencils or the abdominal coremata remains to be studied in detail. Other pyraloid examples of this kind have been discussed by Solis et al. (2009), and Hayden (2010, 2011) has since added examples of independently derived and readily reversed structures from the odontiine crambids: some species of *Cliniodes* bearing tufts on the prothoracic femora and 7^th^ sternite have sister species in which these structures are reduced or absent. In the genus *Dicepolia*, the two most commonly encountered species share a prothoracic tibial tuft but are otherwise unrelated; meanwhile an S7 tuft is an unreversed synapomorphy of Neotropical members of the genus.

Apropos of species diagnosis, we recall that as compelling as are the raw diversity of secondary sexual characters and the demonstrations of their phylogenetic lability, there have been suggestions that the expression of such structures may be underlain by rather simple genetic systems. Following Ohno’s (2000) observation of within-brood polymorphism with respect to mid-tibial tufts in *Ostrinia* (Crambidae), Frolov et al. (2007) suggested that this polymorphism may depend on two di-allelic loci unrelated to reproductive isolation. Although Frolov et al. speculated somewhat with respect to the putative roles of such structures in sympatric speciation, it is clear that even seemingly complex structures may be subject to the simple rules of Mendelian inheritance, character fixation and extinction. If such is the case, then even “important” characters such those involved in courtship, lekking, or mate recognition might be polymorphic, locally fixed, or even frequency-dependent, and might not necessarily serve to diagnose species. Apparent polymorphism in features such as tibial hair pencils (as in *Schacontia lachesis*) or abdominal coremata (as in *Schacontia themis*) speak to the possibility that species pairs such as *Schacontia lachesis*-*atropos* or *Schacontia themis*-*rasa* might be conspecific. Our limited DNA barcode data suggest otherwise for the latter species pair, but can not corroborate the diagnostic power of the morphological characters themselves without more extensive sampling.

## Supplementary Material

XML Treatment for Schacontia

XML Treatment for Schacontia medalba

XML Treatment for Schacontia chanesalis

XML Treatment for Schacontia umbra

XML Treatment for Schacontia speciosa

XML Treatment for Schacontia ysticalis

XML Treatment for Schacontia themis

XML Treatment for Schacontia rasa

XML Treatment for Schacontia nyx

XML Treatment for Schacontia clotho

XML Treatment for Schacontia lachesis

XML Treatment for Schacontia atropos

## Figures and Tables

**Table:** 

	0 10	20	30	40	50	60	
medalba	0011011-01	10110-0001	0001101000	011210-000	1110--010-	-0-10-00-0	-001
chanesalis	0011011-01	10110-0001	0001101000	011210-000	1100--010-	-0-10-0100	-001
umbra	0011011-01	10110-0-01	0001101000	011210-000	1110--010-	-0-?0-----	---1
speciosa	0022011-01	10110-0002	1102101000	011210-000	2100--000-	-1010-1101	0001
ysticalis	1122011-11	00110-0102	1102101111	011110-001	21010-0120	0101101101	0011
themis	1102011010	0101111102	1102101111	1111111111	22010-0120	0101111111	1111
rasa	1102011010	01010-0002	1102101111	0111111111	22010-0120	0101111101	1111
nyx	1102011110	0101100102	1102101111	1111110001	3201111121	1101111111	1111
clotho	1102011010	0101111102	1102101111	1111110001	3201111121	1111111101	1111
lachesis	1102011110	01011*1102	1102101111	1111110101	3201100111	1111111111	1111
atropos	1102011110	01010-0-02	1102101111	0111110001	3201100111	111111----	---1
Glaphyria	1100100010	10000-0111	0010010110	000000-000	00?0--000-	-0-0111?-1	1010
Hellula	1100100-01	00000-0110	0010010110	000000-000	00?0--000-	?0-00-10-1	1010
Eustixia	1111000--1	00-00-0111	0101010110	000000-000	00?0--000-	-0-0101101	1000

## Appendix I

Matrix
